# The Human ABCG1 Transporter Mobilizes Plasma Membrane and Late Endosomal Non-Sphingomyelin-Associated-Cholesterol for Efflux and Esterification

**DOI:** 10.3390/biology3040866

**Published:** 2014-12-04

**Authors:** Edward B. Neufeld, Katherine O’Brien, Avram D. Walts, John A. Stonik, Daniela Malide, Christian A. Combs, Alan T. Remaley

**Affiliations:** 1 Lipoprotein Metabolism Section, Cardiovascular and Pulmonary Branch, National Heart, Lung and Blood Institute, National Institutes of Health, Bethesda, MD 20892, USA; E-Mails: jstonik@verizon.net (J.A.S.); Alan.Remaley@nih.gov (A.T.R.); 2 Lipid Trafficking Core, National Heart, Lung and Blood Institute, National Institutes of Health, Bethesda, MD 20892, USA; E-Mails: obrienk@nhlbi.nih.gov (K.O.); awalts@yahoo.com (A.D.W.); 3 NHLBI Light Microscopy Core Facility, National Institutes of Health, Bethesda, MD 20892, USA; E-Mails: dmalide@mail.nih.gov (D.M.); combsc@nih.gov (C.A.C.)

**Keywords:** ABCG1, cholesterol, cholesterol efflux, vesicular trafficking, HDL, rescretion

## Abstract

We have previously shown that GFP-tagged human ABCG1 on the plasma membrane (PM) and in late endosomes (LE) mobilizes sterol on both sides of the membrane lipid bilayer, thereby increasing cellular cholesterol efflux to lipid surfaces. In the present study, we examined ABCG1-induced changes in membrane cholesterol distribution, organization, and mobility. ABCG1-GFP expression increased the amount of mobile, non-sphingomyelin(SM)-associated cholesterol at the PM and LE, but not the amount of SM-associated-cholesterol or SM. ABCG1-mobilized non-SM-associated-cholesterol rapidly cycled between the PM and LE and effluxed from the PM to extracellular acceptors, or, relocated to intracellular sites of esterification. ABCG1 increased detergent-soluble pools of PM and LE cholesterol, generated detergent-resistant, non-SM-associated PM cholesterol, and increased resistance to both amphotericin B-induced (cholesterol-mediated) and lysenin-induced (SM-mediated) cytolysis, consistent with altered organization of both PM cholesterol and SM. ABCG1 itself resided in detergent-soluble membrane domains. We propose that PM and LE ABCG1 residing at the phase boundary between ordered (L_o_) and disordered (L_d_) membrane lipid domains alters SM and cholesterol organization thereby increasing cholesterol flux between L_o_ and L_d_, and hence, the amount of cholesterol available for removal by acceptors on either side of the membrane bilayer for either efflux or esterification.

## 1. Introduction

Cholesterol plays a critical role in organizing other membrane lipids and proteins into functional units that regulate a variety of membrane-dependent processes including cell signaling, lipid and protein sorting and trafficking, and, cell death. Membrane cholesterol content is maintained by complex cellular homeostatic mechanisms that regulate cholesterol synthesis, receptor-mediated lipoprotein uptake, esterification and storage in lipid droplets, vesicular and non-vesicular-mediated intracellular trafficking between different membrane compartments, and removal from the cell via transfer to extracellular acceptors. A variety of membrane and cytosolic proteins have been shown to be involved in regulating intracellular cholesterol trafficking, including several ABC proteins.

We have previously shown that a HeLa cell line that stably expresses a functional human GFP-tagged ABCG1 transporter, together with its parental, non-ABCG1-expressing HeLa cell line provide a suitable model to study the cellular localization, trafficking and site(s) of function of ABCG1 [1]. These studies established that ABCG1 on the plasma membrane (PM) and late endosomes (LE) enhances cellular cholesterol efflux to extracellular acceptors with a lipid surface, and provided evidence that ABCG1 mobilizes cholesterol on both sides of the membrane lipid bilayer. Previous studies have shown that ABCG1 increases the availability of cholesterol in the outer leaflet of the PM for removal by extracellular lipid acceptors [2,3]. ABCG1 appears to possess different binding sites for sphingomyelin (SM) and cholesterol and its affinity for cholesterol is increased by SM [4]. However, we have shown, using SMase, that ABCG1-mediated cellular cholesterol efflux is not SM-dependent [1], although Sano *et al*., using different methods, have reported it be SM-dependent [5]. The pools of membrane cholesterol available for ABCG1-mediated efflux, and the mechanisms by which ABCG1 renders these pools available for efflux are currently poorly understood. Herein, we have used this heterologous expression system to gain further insights into the site(s) and mechanism(s) underlying enhanced ABCG1-mediated cellular cholesterol efflux.

Cholesterol resides in different membrane pools characterized by different kinetic and thermodynamic properties. Cholesterol is tightly packed in liquid ordered membrane microdomains (L_o_) together with saturated long chain SM, glycosphingolipids, and lipid-anchored proteins [6]. L_o_-cholesterol hydrogen bonds with the sphingosine backbone of sphingolipids and is shielded by their bulky head groups [6,7]. Sphingolipids have been shown to be transiently (~10–20 ms) trapped in cholesterol-mediated molecular complexes dwelling within <20-nm diameter areas [8]. Thus, L_o_ membrane domains are small, highly dynamic structures and moreover, cholesterol rapidly fluxes between L_o_ and L_d_ membrane domains. SM-associated cholesterol immobilized together with the other lipids and proteins in ordered membrane microdomains is resistant to cyclodextrin (CD)-mediated extraction [9,10,11] and solubilization in cold Triton X-100 detergent [12].

Although a great deal of attention has focused on SM-associated cholesterol in L_o_, little is known about the regulation and function of non-SM-associated cholesterol, which resides in loosely packed, disordered liquid membrane regions (L_d_) enriched with unsaturated phosphatidylcholine and other phospholipids [6]. Non-SM-associated cholesterol can freely diffuse within each monolayer, is readily extracted by cyclodextrins, and together with the other lipids and proteins residing in disordered membrane regions, is soluble in cold detergent [9,10,11]. To date, little is known concerning the mechanisms that regulate the movement of cholesterol between L_o_ and L_d_ membrane domains.

In the present study, we examined the cellular pools of cholesterol that are mobilized for efflux and esterification by ABCG1-mediated and non-ABCG1-mediated pathways. We assessed the effect of ABCG1 expression on the distribution of cholesterol and SM in membrane domains, using lipid-specific cytochemical staining methods, cold detergent extraction, and cholesterol- and SM-dependent cytotoxins. In addition, we manipulated membrane SM content using SMase to acutely deplete membrane SM, myriocin to chronically deplete SM, and prolonged incubation with exogenous SM to enrich membrane SM, and then monitored ABCG1-mediated alterations in SM-associated and non-SM-associated cholesterol pool sizes by measuring methyl-β-cyclodextrin (CD)-mediated cellular cholesterol efflux. We also determined the capacity of cholesterol acceptors with a lipid surface, PC liposomes, and HDL, to efflux ABCG1 mobilized *vs*. non-ABCG1 mobilized cellular cholesterol from cells with native or altered SM content.

Consistent with our previous studies, we presently show that the human ABCG1 transporter alters the organization of PM cholesterol and SM, increases the amount of rapidly cycling non-SM-associated cholesterol at the PM and LE, and mobilizes this increased non-SM-associated cholesterol for efflux to extracellular acceptors, or, relocation to intracellular sites of esterification. Our studies suggest that ABCG1 residing at the phase boundary between ordered (L_o_) and disordered (L_d_) membrane lipid domains alters SM and cholesterol organization thereby increasing cholesterol flux between L_o_ and L_d_ and hence, the amount of cholesterol available for removal by either extracellular (efflux) or intracellular cholesterol acceptors (esterification). These findings confirm our previous observation that ABCG1 mobilizes cholesterol on both sides of membrane lipid bilayer. Insofar as excess non-SM-associated membrane cholesterol is potentially cytotoxic [13,14], ABCG1 appears to play a key role in shunting cytotoxic pools of membrane cholesterol into cytoprotective efflux and esterification pathways.

## 2. Experimental Section

Cell Culture—A stably transfected ABCG1-GFP HeLa cell line was established as previously described [1]. Parental and stably expressing ABCG1-GFP HeLa cells were grown in AMEM (Life Technologies, Inc., Grand Island, NY, USA), supplemented with 10% fetal bovine serum, 2 mM glutamine, 100 IU/mL of penicillin, 100 µg/mL streptomycin, and 100 µg/mL G418.

Fluorescent Membrane Lipid Trafficking—To monitor incorporation into the plasma membrane, cells were labeled with 5 µM BODIPY-sphingomyelin(SM)/BSA in HEPES buffer for on ice for 30 min, washed with cold buffer, and then imaged. To monitor intracellular trafficking, cells were labeled with 5 µM BODIPY-SM/BSA in HEPES buffer for on ice for 30 min, washed with cold buffer, and then incubated for 30 min at 37 °C in AMEM/0.1% BSA [15]. To monitor trafficking of intracellular pools of BODIPY-SM to the PM, a portion of labeled cells were then incubated with either 5% defatted BSA in HEPES buffer, or, with buffer alone. Labeled cells were immediately imaged by confocal microscopy using 488 nm excitation, and LP 505 (Red + Green Channel), BP 505-530 (Green Channel) and LP 610 (Red Channel) filters to detect fluorescent emissions. The “green” BODIPY-SM signal far exceeded the GFP signal allowing the gain to be set such that the GFP fluorescent signal from ABCG1-GFP cells that were not labeled with BODIPY-SM contributed no signal to the image. Ratio fluorescence images were rendered using Zeiss 510 software, such that each pixel represents the intensity of the Red Channel/Green Channel fluorescence intensity. Relative pixel intensity was rendered as a color scale using LUTs (Imaris Software). For labeling GM1 ganglioside, living cells were incubated with 100 ng/mL Alexa594-tagged cholera toxin B in AMEM/0.1% BSA on ice for 20 min, and then chased with AMEM buffer medium supplemented with 10% fetal bovine serum, 2 mM glutamine, 100 IU/mL of penicillin, 100 µg/mL streptomycin for 0, 10, 20, or 60 min, washed and fixed before imaging. To visualize endogenous pools of PM SM, cells were incubated with 1 µg/mL lysenin in 1% BSA/PBS for 30 min on ice, washed, fixed and then immunostained with lysenin-specific antibodies using the protocol of Ishitsuka *et al*. [16]

Evaluation of detergent-resistant membrane lipids—For biochemical analyses, nearly confluent cells were labeled with 1 µC/mL ^3^H-cholesterol for 24 h in AMEM/10% FBS medium, washed with PBS, and chilled on ice with fresh PBS, and then incubated with either ice cold PBS or ice cold 1% TX-100 for 5 min. The medium was centrifuged and the supernatant removed and counted. The combined cell pellet and cells were extracted with hexane:isopropanol (3:2), combined and counted. The percent detergent-resistant cholesterol was calculated as (total cell dpm/total cell dpm + supernatant dpm) × 100. For microscopic analyses, cells plated on glass slides were washed with PBS, incubated at 37 °C in the presence or absence of 0.1 U/mL neutral sphingomyelinase (from *Staphylococcus* *aureus*, Sigma, St. Louis, MO, USA) for 30 min and then washed with PBS. Cells were then pre-chilled on ice in PBS, and incubated with PBS alone or PBS containing 1% TX-100 detergent for 30 min. After washing with ice cold PBS, cells were fixed with 3% paraformaldehyde for 10 min, washed with cold PBS, stained with 0.5% filipin for 30 min, or, lysenin, washed and imaged by confocal microscopy. For phospholipid analysis, nearly confluent cells were labeled with 1 µCi/mL ^3^H-methyl-choline for 24 h, washed with PBS, and then incubated at 37 °C in the presence or absence of 0.1 U/mL neutral sphingomyelinase washed and cell-associated lipids were extracted with hexane:isopropanol (3:2).

Confocal Microscopy—Laser scanning confocal microscopy to monitor GFP, filipin and Alexa 568 and Alexa 594 fluorescence, was performed using a Zeiss 510 LSCM. 3D imaging was performed as previously described [1,17]. All microscopy experiments were repeated at least three times. In each experiment, imaging parameters were optimized the for the cell treatment that produced maximal signal intensity, to avoid pixel saturation. All imaging for the rest of the treatment groups was performed using the identical parameters, thereby allowing comparison of the relative distribution and intensity of labeling in subcellular compartments between the different cell types and different treatments. For filipin fluorescence, imaging conditions were optimized as above using one set of slides, and then images were obtained using a second set of unexposed slides. Focusing of both control and ABCG1-GFP cells was performed using GFP excitation/emission so as not to pre-bleach the filipin fluorescence. For control cells, the laser power and gain was optimized to allow the use of control cell autofluorescence in the GFP channel to focus.

Lipid Efflux Assays—HDL subfractions, LDL, and apoA-I were obtained from human serum by ultacentrifugation, as previously described [17,18]. Liposomes were prepared by sonication of L-α-phosphatidylcholine (Avanti Polar Lipids) in PBS, as previously described [19]. For cellular lipid efflux studies, all assays were conducted using six replicates (wells), and represent a minimum of three experiments. For cholesterol efflux, nearly confluent cells in 24-well plates cells were labeled with 1 µC/mL ^3^H-cholesterol for 24 h in AMEM/10% FBS medium, washed, and then incubated for 4 h in AMEM containing 1 mg/mL of bovine serum albumin in the presence or absence of HDL, (25, 50, or 100 µg/mL) or, PC liposomes (25, 50, or 100 µg/mL). For acute SM-depletion studies, cells were incubated in the presence or absence of 0.1 U/mL neutral sphingomyelinase (from *Staphylococcus* *aureus*, Sigma) in AMEM/0.1% BSA medium in the presence or absence of acceptors, as described. For chronic SM-depletion and chronic SM-supplementation studies, cells were incubated in the absence or presence of 1 µM myriocin (Sigma), or 40 µM bovine sphingomyelin (Sigma), respectively, during ^3^H-cholesterol labeling. Efflux of cellular ^3^H-cholesterol to methyl-β-cyclodextrin in HEPES buffer at the indicated concentrations was performed at either 4 °C for 10 min, or 37 °C for 30 min, to assess plasma membrane and cellular pools of cholesterol, respectively. Lipid-associated ^3^H extracted into hexane:isopropanol (3:2) was analyzed by scintillation counting. The percentage of cellular lipid that effluxed was calculated by dividing the medium counts by the sum of the radioactive counts in the medium plus the cell fraction, after correction for efflux to 0.1% BSA medium.

Cholesterol Esterification—Cells labeled with ^3^H-cholesterol were incubated in the absence or presence of 0.1 U/mL neutral sphingomyelinase (from *Staphylococcus* *aureus*, Sigma) in AMEM/0.1% BSA for 30 min at 37 °C, washed and then incubated in the presence or absence of either 10 mM methyl-β-cyclodextrin for 10 min at 37 °C, or, 50 mg/mL HDL for 4 h, and then cellular ^3^H-sterols were extracted and separated by thin-layer chromatography and cholesterol and cholesterol ester radioactivity was analyzed as previously described [1].

Cytolytic Assays—Amphotericin B and lysenin cytolytic assays were based on the method used by Jacobs *et al*. [20], and Hanada *et al*. [21], respectively. Briefly, control and ABCG1 cells grown for 2 d in 96-well plates were then treated (eight replicates) with either 100 µL of medium containing 0–100 µg/mL amphotericin B for 5 h, or, 0–50 ng/mL lysenin for 30 min at 37 °C, washed with PBS, incubated with 2.5 mg/mL MTT (to label dead cells) for 2 h, and washed. After addition of 50 µL DMSO, the absorbance at 570 nm for each well was assessed using a spectrophotometric plate reader. The absorbance values were corrected by subtraction of the value obtained from the background control, and the corrected values were used as a measure of the viability of cells, expressed relative to untreated control cells as 100%.

## 3. Results and Discussion

### 3.1. Cholesterol at the PM and LE of ABCG1-Cells Markedly Increases Incorporation of Exogenous Fluorescent SM, a Marker for Non-SM-Associated Cholesterol

**Figure 1:**
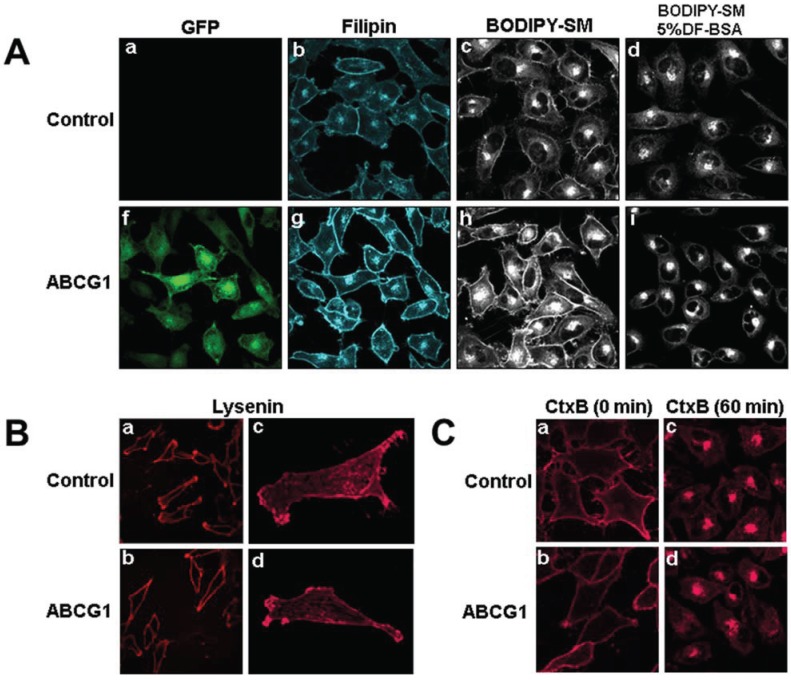
(**A**) ABCG1-GFP expression increases the amount of rapidly recycling, non-SM-associated cholesterol at the PM and LE. Cells were labeled with filipin, BODIPY-SM and Alexa594-CtxB, and imaged as described in “Experimental Section.” (a,f: GFP fluorescence; b,g: filipin; c,d,h,i; BODIPY-SM fluorescence). (**B**) ABCG1 expression does not alter SM-specific cytochemical lysenin staining at the PM. Note similar lysenin PM staining in single confocal image slices (a, b) as well as in 3D maximum projection images (c, d) of control and ABCG1 cells, respectively. (**C**) Cell surface GM_1_ content and trafficking is not altered by ABCG1. The amount and distribution of fluorescent-tagged CtxB in living control (a, c) and ABCG1 cells (b, d) after labeling in the cold (a, b) and incubation for 60 min at 37 °C (c, d) is similar. Note that the imaging conditions in (a) and (f) were optimized for GFP fluorescence. The GFP fluorescence was exceeding low compared to the “green” BODIPY-SM signal and, the contribution of GFP to the “green” fluorescence in (c), (d), (h) and, (f) was essentially eliminated by adjusting the gain such that the GFP fluorescent signal from ABCG1 cells that were not labeled with BODIPY-SM was essentially eliminated (Supplemental Figure 1).

As we have previously shown [1], ABCG1-GFP-containing membranes at the cell surface and in LE are enriched with cholesterol compared to control cells, as revealed by cytochemical staining of cholesterol with filipin (Figure 1A). This increase in filipin staining may represent an increase in cholesterol content or, an increase in filipin-accessible pools of cholesterol.

BODIPY-sphingolipids have been used as a probe to report elevated membrane cholesterol levels in cholesterol-enriched LE membranes in human lysosomal storage diseases [22] and Tangier disease fibroblasts [23]. Fluorescent SM incorporates into detergent-soluble membranes and colocalizes with markers of disordered membrane regions [24]. BODIPY-SM serves as a marker for non-SM-associated cholesterol, presumably because it interacts with cholesterol not already associated with endogenous SM. BODIPY-SM reports its packing density in membranes as green and red fluorescence when BODIPY-SM is present at low and high mole fractions, respectively [25]. Previous studies by Pagano *et al.* have established that the ratio of fluorescence intensities in liposome preparations is linear from 2 mol to 50 mol % BODIPY-SM [26,27]. We tested whether the incorporation and intracellular trafficking of exogenously supplied fluorescent BODIPY-SM would be altered in the PM and LE of ABCG1 cells. Compared to control cells, ABCG1 cells demonstrated markedly increased uptake of BODIPY-SM at 4 °C into the PM, as monitored by ratio fluorescence imaging of red:green fluorescence (Figure 5A, Supplemental Figure 2). To test whether the increased PM and LE cholesterol in ABCG1 cells alters intracellular trafficking of BODIPY-SM, the PM of control and ABCG1-GFP cells were labeled on ice with 5 µM BODIPY-SM, washed, and then warmed for 30 min at 37 °C to allow the fluorescent SM to traffic to intracellular compartments. As shown in Figure 1, ABCG1 cells demonstrated markedly increased retention of BODIPY-SM in the PM and LE. In control cells after the 30 min warm-up, BODIPY-SM was present in cellular membranes at a low mole fraction (Figure 1; Supplemental Figure 3) and trafficked from the PM to the Golgi, and to a lesser extent the to the ER; only a small amount of fluorescent SM was present on the cell surface. After the 30 min warm-up, in marked contrast to control cells, BODIPY-SM was retained in the PM of ABCG1 cells as both high (red fluorescence) and low mole (green fluorescence) fractions (Figure 1; Supplemental Figure 3). In ABCG1 cells, BODIPY-SM in perinuclear LE was present mostly at a low mole fraction (green fluorescence), and to a lesser extent at a high mole fraction (red fluorescence). The retention of BODIPY-SM in the ABCG1 PM and LE greatly exceeded that in control cells, at both high and low mole fractions.

To determine if BOPIPY-SM retained in ABCG1 LE can cycle back to the cell surface, we incubated cells that had been labeled and chased (30 min) as above, with defatted (DF)-BSA at 37 °C for 3-min (Figure 1 and Supplemental Figure 4). DF-BSA removed the little fluorescent SM that was present at the PM of control cells. DF-BSA removed nearly all the fluorescent SM retained at the PM and LE of ABCG1 cells. These findings suggest that fluorescent SM retained by membrane cholesterol in ABCG1 cells rapidly cycles between LE and the PM. BODIPY-SM in the Golgi and ER in either cell line did not appreciably cycle back to the cell surface during this time. These findings are consistent our initial observation that pools of filpin-accessible cholesterol accumulates at the PM and LE of ABCG1 cells [1] and suggests that this cholesterol is a non-SM-associated pool that rapidly cycles between these sites.

We next assessed the effect of ABCG1 expression on PM SM content by confocal microscopic assessment of immunostaining of the SM-specific binding toxin lysenin. The amount and distribution of endogenous SM at the PM was not altered by ABCG1 (Figure 1B), suggesting that the amount of SM-associated-cholesterol was not altered by ABCG1. To further assess the effect of ABCG1 on the distribution and trafficking of lipids in ordered microdomains, we monitored the intracellular trafficking of the ganglioside GM_1_ in living cells, using the GM_1_-specific marker, fluorescent cholera toxin B (CtxB) [28]. SM-enriched microdomains have previously shown to be spatially and functionally distinct from GM_1_-enriched microdomains [29]. As seen in Figure 1C and Supplemental Figure 5, ABCG1 did not alter either the amount of CtxB binding to the PM, or the trafficking of PM-derived CtxB to endosomes and the Golgi. Thus, ABCG1 did not alter the content or trafficking of PM GM_1_ or endogenous SM, two different lipids residing in different ordered microdomains. Taken together these findings suggest that retention of exogenous fluorescent SM at the cell surface and endosomes of ABCG1 cells (Figure 1A), does not involve perturbed trafficking of cholesterol in ordered lipid microdomains, but rather is likely due to retention by altered non-SM-associated cholesterol at these sites.

### 3.2. ABCG1-GFP Increases PM and LE Non-SM-Associated Detergent-Soluble Cholesterol as Well as PM Non-SM-Associated Detergent-Resistant Cholesterol

**Figure 2:**
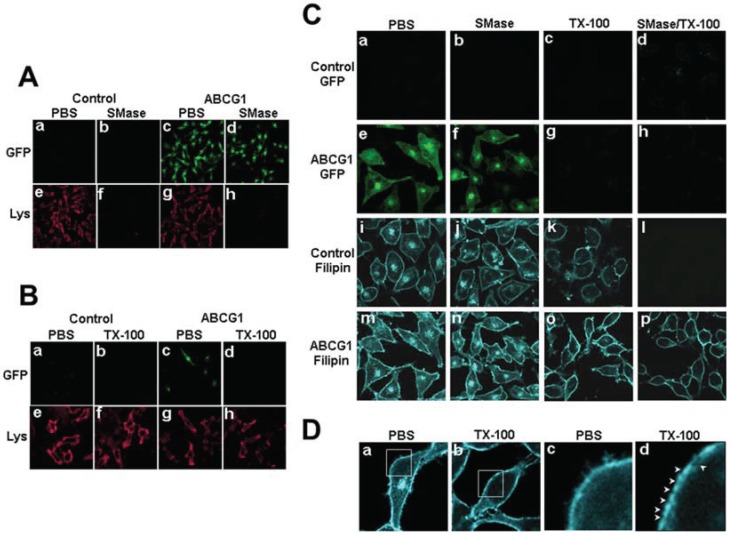
ABCG1-GFP alters cholesterol detergent solubility. (**A**) Control and ABCG1 cells were incubated with PBS or SMase, washed, fixed and immunostained with lysenin as described in “Experimental Section”. (**B**) Control and ABCG1 cells were incubated with cold PBS or 1% TX-100 detergent for 30 min, washed, fixed and immunostained with lysenin as described in “Experimental Section”. (**C**) Control and ABCG1 cells pre-treated with or without SMase, were extracted with 1% TX-100 on ice for 30 min, fixed, filipin stained, and imaged by confocal microscopy as described in “Experimental Section.” (**D**) Higher magnification of filipin-stained AGCG1 cells clearly reveals that LE FC (a) is removed by TX-100 (b). Enlargements of the boxed regions in (a) and (b), shown in (c) and (d), respectively, reveal that TX-100 treatment depleted FC from PM domains (*arrowheads*).

Cholesterol that is soluble in cold detergent represents more highly mobile pools of cholesterol, whereas cholesterol that remains after cold detergent extraction resides in membrane microdomains in association with sphingolipids or saturated phospholipids [6]. To better understand the nature of the pools of excess cholesterol at the PM and LE of ABCG1 cells, living control and ABCG1 cells were extracted with cold 1% Triton X-100 and the amount and cellular distribution of cholesterol was assessed. Extraction of ^3^H-cholesterol from control and ABCG1 cells with cold Triton-X 100 demonstrated that ABCG1 expression did not alter the percent cell-associated detergent-resistant cholesterol (69.5 ± 2.96 and 69.2 ± 3.07, mean ± S.D., for control and ABCG1 cells, respectively; *p* = 0.8578, unpaired two-tailed *t*-test), suggesting that the amount of L_o_ cholesterol was not altered. Cold detergent extraction had little if any effect on lysenin staining (Figure 2B), consistent with the retention of SM in detergent-resistant membrane domains in both control and ABCG1 cells. Taken together these findings suggest that ABCG1 does not alter the amount of SM-associated cholesterol.

As shown in Figure 2C and Supplemental Figure 6, confocal microscopy revealed that virtually all of the intracellular cholesterol as well as a portion of cholesterol on the PM were removed by cold detergent extraction in both control and ABCG1 cells (Figure 2D). Thus, the excess filipin-accessible intracellular cholesterol in ABCG1 cells represents detergent-soluble, mobile pools of cellular cholesterol. Interestingly, since ABCG1 itself was detergent-soluble (Figure 2), ABCG1 on the PM and LE appears to be associated with detergent-soluble pools of cholesterol (Figure 2). Compared to control cells, ABCG1 cells were also observed to have increased detergent-resistant cholesterol on the PM (Figure 2C, o *vs*. k). To further assess the role of SM in forming detergent-resistant pools of cholesterol, living cells were incubated at 37 °C in the absence or presence of sphinomyelinase (SMase) to deplete cellular SM prior to extraction with cold Triton X-100. SMase treatment reduced cellular SM metabolically labeled with ^3^H-methyl-choline by 70% in both ABCG1 and control cells and markedly reduced cell surface SM-specific lysenin staining (Figure 2A). A portion of this excess detergent-resistant PM cholesterol in ABCG1 cells does not appear to be associated with SM insofar as SMase pre-digestion of cellular SM did not render this cholesterol detergent-soluble (Figure 2C, p *vs*. o). Since neither the percent of detergent-resistant cholesterol nor the amount of SM (Figure 1A) or detergent-resistant SM (Figure 1B) and thus, cholesterol-associated SM, was altered by ABCG1 expression, it would appear that in ABCG1 cells the amount of non-SM-associated detergent resistant and non-SM-associated cholesterol were increased to a similar extent. Taken together these findings suggest that cholesterol on the PM and LE of ABCG1 cells that is available for efflux does not appear to be associated with endogenous SM and that a portion of ABCG1-modified cholesterol at the cell surface resides in non-SM-associated, detergent-resistant membrane.

#### 3.2.1. ABCG1-GFP Expression Increases PM and LE Pools of Non-SM-Associated Cholesterol Available for Efflux to CD

We assessed ABCG1-induced changes in the amount of cellular cholesterol in SM-associated and non-SM-associated pools by monitoring cellular ^3^H-cholesterol efflux to methyl-β-cyclodextrin (CD) at 4 °C and 37 °C (to assess PM, and, total cellular cholesterol pools, respectively), in the presence or absence of SMase. Acute SM depletion via SMase-mediated hydrolysis to ceramide converts SM-associated PM cholesterol (L_o_) from a pool that is slowly depleted by CD, into a rapidly CD-depletable, non-SM-associated colesterol pool (L_d_) [9]. Consistent with this, recent molecular dynamic simulations suggest that CD removes cholesterol from L_d_ and that cholesterol migrates to L_d_ from L_o_ as cholesterol is depleted from L_d_ [11]. The size of the SMase-mobilized pool of cellular cholesterol is calculated as the difference between the percent cellular cholesterol efflux to CD in SMase-treated *vs*. non-SMase-treated cells [9].

**Figure 3:**
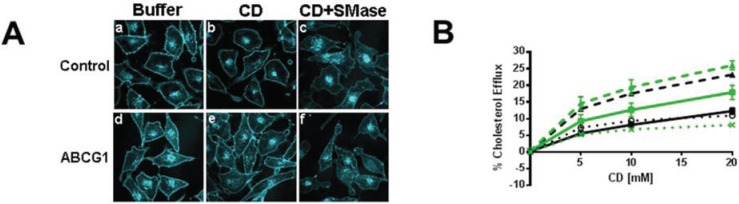

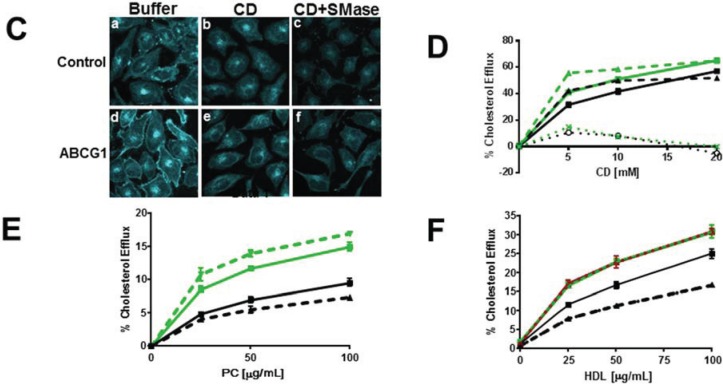
ABCG1-GFP expression increases efflux of SMase-mobilized cholesterol from the PM and LE. (**A**) Cold CD depletes excess PM FC from living ABCG1 cells. Control (a–c), and ABCG1 (d–f) cells pre-treated without (a,b,d,e), or, with SMase (c,f), were FC-depleted using 20 mM CD for 1h at 4 °C (b,c,e,f), then stained with filipin. (**B**) ABCG1 increases PM non-SM-associated-FC. Control (*black*) and ABCG1 (*green*) cells labeled with ^3^H-FC were incubated in the absence or presence of SMase (solid and dashed lines, respectively) and, the percent of total cellular FC effluxed to CD for 1 h at 4 °C was determined. (**C**) Warm CD depletes excess ABCG1-induced PM and LE FC. (**C**) Control (a–c), and ABCG1 (d–f) cells pre-treated without (a,b,d,e), or, with SMase (c,f), were incubated with 5 mM CD for 10 min at 37 °C. (**D**) ABCG1 increases non-SM-associated cellular FC. Control (*black*) and ABCG1 (*green*) cells labeled with ^3^H-FC were incubated in the absence or presence of SMase (solid and dashed lines, respectively) and, the percent of total cellular FC effluxed to CD for 10 min at 37 °C was determined. (**E**) ABCG1 enhances efflux of SMase-mobilized FC to liposomes and (**F**) HDL. Control (*black*) and ABCG1 (*green*) cells labeled with ^3^H-FC were incubated in the absence or presence of SMase (solid and dashed lines, respectively) for 4 h and, the percent of total cellular FC effluxed to liposomes or HDL was determined. For (**B**, **D**) PM SM-associated FC is represented by the difference curves (percent FC efflux of (SMase-treated cells)—(non-treated cells)); *black* and *green* *dotted* lines, control and ABCG1 cells, respectively). All values are expressed as mean + S.D. Data shown is representative of at least three replicate experiments Two-way ANOVA analyses using multiple comparisons revealed that ABCG1 expression and SMase treatment significantly increased the percent CD-mediated cholesterol efflux in (**B**) and (**D**) *p* < 0.0001. However, the values for the percent cellular efflux for SMase-treated control and SMase-treated ABCG1 cells (dashed lines) using 15 mM and 10 mM CD were not significantly different in (**B**) (*p* = 0.1592 and *p* = 0.0841, respectively), or in (**D**) (*p* = 0.2904 and *p* = 0.4282, respectively). In addition the value for the percent Cholesterol Efflux was not significantly different for SMase-treated ABCG1 cells *vs*. non-treated ABCG1 cells using 20 mM CD (*p* = 0.9988). All values in (**E**) were significantly different (*p* < 0.0001) with the exception of ABCG1 cells *vs*. SMase-treated ABCG1 cells.

PM cholesterol efflux to CD at 4 °C was enhanced by ABCG1 (Figure 3). As seen in Figure 3A and Supplemental Figure 7, CD extraction at 4 °C reduced the amount of filipin-accessible cholesterol at the PM in both control and ABCG1 cells. SMase treatment concurrent with CD further reduced PM cholesterol. Compared to control cells, the net reduction of cell surface cholesterol by CD alone, or, in combination with SMase appeared to be greater in ABCG1 cells. As seen in Figure 3B, ABCG1 increased the pool of CD-extractable PM ^3^H-cholesterol, with or without SMase pre-treatment. Interestingly, the SMase-induced net increase in CD-mediated cellular cholesterol efflux at 4 °C was similar in both ABCG1 and control cells, suggesting that the amount of SM-associated-cholesterol at the PM is similar in ABCG1 and control cells. Thus, ABCG1 appears to increase the non-SM-associated pool of PM ^3^H-cholesterol available to efflux to CD at 4 °C. These findings are consistent with our observations that ABCG1 increases the amount of filipin-accessible cholesterol in the PM (Figure 1 and Figure 2). Taken together, these findings indicate that ABCG1 increases mobile pools of non-SM-associated-cholesterol at the PM.

Cellular cholesterol efflux to CD at 37 °C was also enhanced by ABCG1 (Figure 3C, D). As seen in Figure 3C and Supplemental Figure 8, CD at 37 °C reduces both intracellular and PM pools of cholesterol, and, SMase in combination with CD further reduces PM and intracellular cholesterol in both control and ABCG1 cells. The net reduction of cellular cholesterol by CD alone, or, in combination with SMase appeared to be greater in ABCG1 compared to control cells. Cellular ^3^H-cholesterol efflux to CD at 37 °C was enhanced by ABCG1 in non-SMase treated cells, at all concentrations of CD (Figure 3D). This finding suggests that ABCG1 increases CD-accessible cholesterol at the PM as well as in intracellular pools that can reach the PM. Conversion of SM-associated cholesterol to non-SM-associated cholesterol via SMase-mediated SM hydrolysis increased CD-mediated cellular cholesterol efflux to a similar extent in both control and ABCG1 cells (Figure 3D), suggesting that the amount of SM-associated-cholesterol is similar in ABCG1 and control cells. CD-mediated cellular cholesterol efflux was still greater in ABCG1 compared to control cells with SMase pre-treatment, consistent with an ABCG1-mediated increase in mobile pools of cholesterol at PM and in intracellular pools that can reach the PM.

#### 3.2.2. SMase Hydrolysis of Cellular SM Increases ABCG1-Mediated Cellular Cholesterol Efflux to Extracellular Lipid Acceptors

We next assessed the effect of reducing cellular SM levels with SMase on ABCG1-mediated cellular cholesterol efflux to extracellular acceptors with a lipid surface. We [1], and others [30,31], have reported that ABCG1 expression enhances cellular cholesterol efflux to PC vesicles, thereby establishing a lipid surface as the minimal requirement for an extracellular acceptor of ABCG1-mobilized cellular cholesterol. As shown above, expansion of cellular pools of non-SM-associated ^3^H-cholesterol via SMase hydrolysis of membrane SM increased CD-mediated efflux in control cells (Figure 3D). Surprisingly, SMase decreased liposome-mediated cellular cholesterol efflux in control cells (Figure 3E). In marked contrast, SMase enhanced liposome-mediated cellular cholesterol efflux in ABCG1 cells (Figure 3E), consistent with ABCG1-mediated mobilization of non-SM-associated cholesterol for efflux to PC liposomes. Comparison of liposome-mediated cholesterol efflux in SMase-treated ABCG1 *vs*. SMase-treated control cells reveals a significant enhancement of non-SM-associated cholesterol efflux in ABCG1 cells. Since ABCG1 expressing cells can efflux by both ABCG1-mediated and non-ABCG1-mediated pathways, these findings suggest that non-SM-associated cholesterol is diverted from the non-ABCG1 to the ABCG1-mediated efflux pathway in ABCG1 cells. Taken together, these findings suggest that ABCG1 mobilizes non-SM-associated cholesterol for efflux to an extracellular cholesterol acceptor with a lipid surface.

We next determined the effect of acute SM depletion on ABCG1-mediated cellular ^3^H-cholesterol efflux to HDL (Figure 3F). SMase also decreased cellular cholesterol efflux to HDL in control, but not, ABCG1 cells (Figure 3F). Compared to control cells, however, ABCG1 markedly increased HDL-mediated cholesterol efflux after SMase-mediated depletion of cellular SM. Thus, ABCG1 appears to mobilize SMase-generated, non-SM-associated-cholesterol for efflux to both PC vesicles and HDL. These studies confirm our previous observation that that PM SM is not required for ABCG1-mediated cellular cholesterol efflux to HDL [1].

#### 3.2.3. ABCG1-Mediated Increased Cellular Cholesterol Esterification is Enhanced by SMase-Mediated Cholesterol Mobilization and is Reduced by CD and HDL

**Figure 4:**
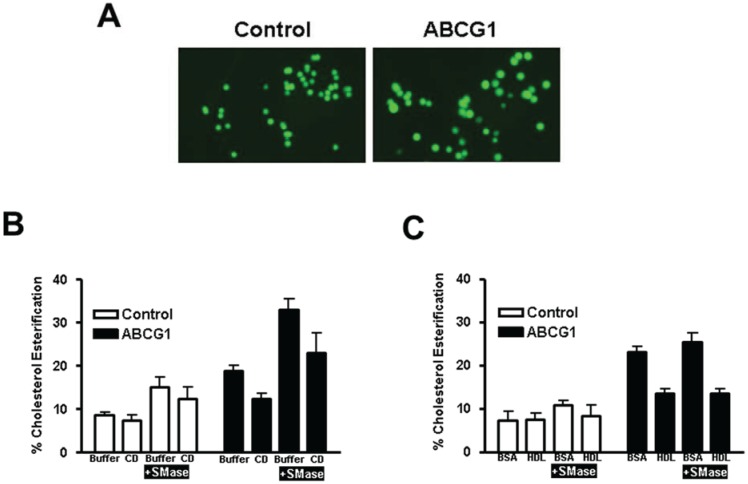
ABCG1 enhances cholesterol esterification. (**A**) Confocal microscopic 3D reconstruction of Nile Red-stained cytoplasmic lipid droplets (maximum projection images). Control and ABCG1 cells labeled with ^3^H-FC pre-incubated with or without SMase were then incubated in buffer alone, or, with (**B**) 10 mM (10 min at 37 °C), or, (**C**) 50 µg/mL HDL (4 h at 37 °C), and ^3^H-cholesteryl ester (CE) formation was monitored. All values are expressed as mean + S.D. Data shown is representative of at least three replicate experiments. Two-way ANOVA analyses using multiple comparisons revealed in (**B**) that in control cells, compared to buffer treatment alone, the percent cholesterol esterification was not significantly different with SMase treatment alone (*p* = 0.0765) or, with CD in the absence (*p* = 0.997) or, presence of SMase (*p* = 0.5975). In ABCG1 cells, compared to buffer treatment alone, the percent cholesterol esterification was significantly increased by SMase treatment (*p* < 0.0001), but not by CD in the absence (*p* = 0.0704) or presence (*p* = 0.0655) of SMase. CD significantly decreased the percent cholesterol esterification in SMase-treated ABCG1 cells (*p* = 0.0023). Compared to control cells in buffer alone, ABCG1 expression significantly increased the percent cholesterol esterification in buffer alone (*p* = 0.0018) and, with SMase treatment in the absence or presence of CD (*p* < 0.0001). In (**C**), two-way ANOVA analysis revealed that in control cells, compared to buffer treatment alone, the percent cholesterol esterification was unaltered by SMase treatment alone (*p* < 0.2142) or, by HDL in the absence (*p* < 0.9999) or, presence of SMase (*p* = 0.9936). In ABCG1 cells, compared to buffer treatment alone, the percent cholesterol esterification was significantly decreased by HDL in the absence or, presence of SMase (*p* < 0.0001). Compared to control cells in buffer alone, ABCG1 expression significantly increased the percent cholesterol esterification in buffer alone (*p* < 0.0001), with HDL (*p* < 0.0049) and, with SMase treatment in the absence (*p* < 0.0001) or, presence of HDL (*p* < 0.0052).

We assessed the effect of ABCG1 on cellular cholesterol esterification by cytochemical and biochemical analyses. As seen in Figure 4A and Supplemental Figure 9, confocal microscopic imaging of the cytoplasm of Nile Red-stained cells reveals increased lipid droplet size and intensity concomitant with ABCG1 expression. To gain insight into the mechanisms involved in ABCG1-mediated cholesterol esterification, we monitored the effect of SMase pre-treatment with, or, without subsequent CD- or HDL-mediated extraction of cellular cholesterol, on ^3^H-cholesteryl ester formation in control and ABCG1 cells labeled with ^3^H-cholesterol. As seen in Figure 4B, ABCG1 expression alone markedly enhanced cellular cholesterol esterification. In contrast to control cells, cholesterol esterification in ABCG1 cells was markedly enhanced by SMase digestion and, substantially reduced by either CD-mediated (Figure 4B), or, HDL-mediated (Figure 4C) efflux of cellular cholesterol. Our findings are consistent with a previous report that the ABCG1-mediated enhancement of cellular esterification is decreased by HDL [2].

**Figure 5:**
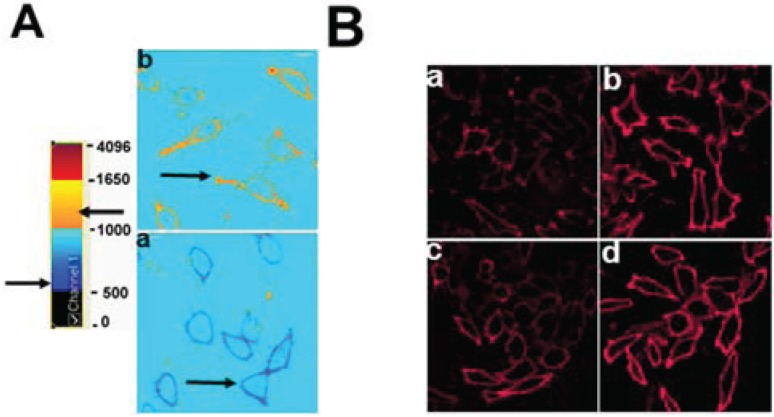

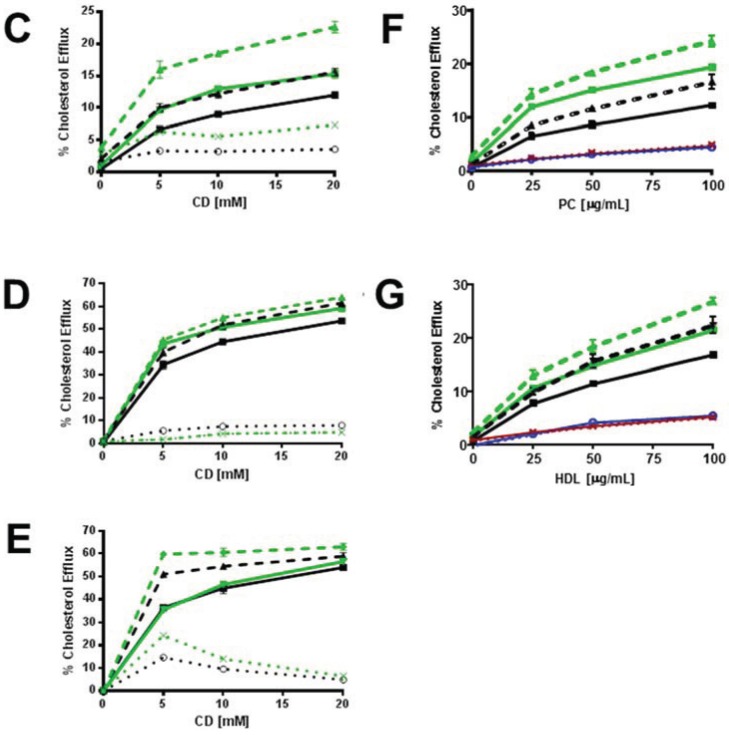
ABCG1 increases SM-enhanced cholesterol efflux. (**A**) Ratio fluorescence (red:green) of BODIPY-SM incorporation into control (a) and ABCG1 (b) PM. **(B)** Exogenous SM enriches control and ABCG1 PM SM. Control and ABCG1 cells treated without (a) and (c), respectively, or with exogenous SM, (b) and (d), respectively, were stained with SM-specific lysenin toxin. In (**C**–**F**) control and ABCG1 cells incubated in the absence (*solid black* and *green lines*, respectively) or presence (*dashed black* and *green lines*, respectively) of exogenous SM were labeled with ^3^H-FC and then incubated with CD for 1 h at 4 °C (**C**), or for 10 min at 37 °C (**D**), or, with PC liposomes (**F**), or HDL (**G**). (**C**) SM-induced increase in PM non-SM-associated FC is enhanced by ABCG1. Difference curves represent (percent cholesterol efflux of (SM-treated cells)—(non-SM-treated cells)), *dotted lines*, control and ABCG1, *black* and *green* respectively.) (**D**) SM-induces increased cellular FC. Difference curves as in (**C**). (**E**) SM-induced increase in SM-associated-FC pool is enhanced by ABCG1. Control and ABCG1 cells treated with exogenous SM were incubated with (*dashed black and green lines,* respectively), or, without (*solid black and green lines,* respectively) SMase prior to CD-mediated FC efflux at 37 °C, as described above. Difference curves represent (percent cholesterol efflux of (SMase-treated cells)—(non-SMase-treated cells), *dotted lines*; control and ABCG1, *black* and *green,* respectively.) ABCG1 increases SM-enhanced FC efflux to PC liposomes (**F**) and to HDL (**G**). For (**F**) and (**G**) control and ABCG1 difference curves were calculated as in (**C**) in are *blue* and *red*, for control and ABCG1 cells, respectively, in. All values are expressed as mean ± S.D. Data shown is representative of at least three replicate experiments. Two-way ANOVA analyses using multiple comparisons revealed in (**C**) that all values for cold CD-mediated efflux are significantly different (*p* < 0.0001), except for control cells treated with exogenous SM (dashed black line) *vs*. untreated ABCG1cells (green solid line), *p* = 0.7632, *p* = 0.1093, and *p* = 0.8572, for 5, 10 and 20 mM CD, respectively. In (**D**), all values for warm CD-mediated efflux are significantly different (*p* < 0.0001), except for control cells treated with exogenous SM (dashed black line) *vs*. untreated ABCG1cells (green solid line), *p* = 0.0419 and *p* = 0.3274, for 5 and 10 mM CD, respectively. In (**E**), all values for warm CD-mediated efflux are significantly different (*p* < 0.0001), except for control cells treated with exogenous SM (solid black line) *vs*. ABCG1 cells treated with exogenous SM (green solid line), *p* = 0.6892 and p = 0.1857, for 5 and 10 mM CD, respectively. In (**F**), all values are significantly different (*p* < 0.0001). In (**G**), all values are different except for control cells treated with exogenous SM (dashed black line) *vs*. untreated ABCG1cells (green solid line), *p* = 0.1991, *p* = 0.1715, and *p* = 0.1319, for 25, 50 and 100 µg/mL HDL, respectively.

### 3.3. Enrichment of Membrane SM with Exogenous SM Increases ABCG1-Mediated Cellular Cholesterol Efflux

As shown in Figure 5A, ratio fluorescence imaging of control and ABCG1 cells reveals a marked (2-fold) increase in incorporation of exogenously supplied BODIPY-SM at 4 °C into the PM of ABCG1 cells, shown earlier (Figure 1A) to be due to altered pools of PM non-SM-associated-cholesterol. We further probed the role of membrane SM in ABCG1-induced changes in cellular cholesterol distribution by pre-loading cells with exogenous SM to increase membrane SM content, and then measured cellular ^3^H-cholesterol CD-mediated efflux at 4 °C and 37 °C. As revealed by SM-specific immunostaining with lysenin (Figure 5B), incubation of cells with 40 µM SM for 1d increased SM content at the cell surface in both control and ABCG1 cells. Enrichment of cells with SM enhanced cholesterol efflux from control and ABCG1 cells to CD at 4 °C (Figure 5C), and the SM-induced enhancement in efflux was greater in ABCG1 cells. Thus, exogenous SM expanded non-SM-associated PM cholesterol available to efflux to CD in both control and ABCG1 cells, and the expansion in ABCG1 cells was greater. Cellular ^3^H-cholesterol efflux to CD at 37 °C was enhanced in SM-enriched cells (Figure 5D), and the extent of enrichment corresponded to the increase in cholesterol at the PM observed in Figure 5C.

We assessed the expansion of CD-accessible SM-associated-cholesterol pools by exogenous SM in control and ABCG1 cells by again using SMase to acutely reduce cellular SM. As shown in Figure 5E, SM-enrichment expanded the pool of SM-associated-cholesterol to a greater extent in ABCG1 cells.

We next tested the effect of expanding cellular SM levels on cellular cholesterol efflux to extracellular acceptors with a lipid surface. Enhancement of cellular SM content increased ^3^H-cholesterol efflux from both control and ABCG1 cells to a similar extent using either PC liposomes (Figure 5F) or HDL (Figure 5G) as extracellular cholesterol acceptors. SM-enrichment further enhanced ^3^H-cholesterol efflux to PC liposomes and to HDL in ABCG1 cells, compared to both non-treated ABCG1 cells and, SM-treated control cells.

Taken together these findings reveal that SM enrichment increases both SM-associated-cholesterol and non-SM-associated cholesterol in control and ABCG1 cells and, that ABCG1 expression expands both of these pools to a greater extent than control cells. In control cells, exogenous SM increased the total amount of cellular cholesterol available to efflux to CD, PC liposomes and HDL at 37 °C, presumably because of the increased size of the L_o_-cholesterol pool available to replete L_d_ cholesterol that is depleted by the extracellular cholesterol acceptors. ABCG1 further increased cellular cholesterol efflux to these extracellular acceptors, presumably by enhancing the flux of membrane cholesterol between L_o_ and L_d_ pools.

### 3.4. Myriocin-Mediated Reduction of Cellular SM Increases Cellular Cholesterol Efflux

**Figure 6:**
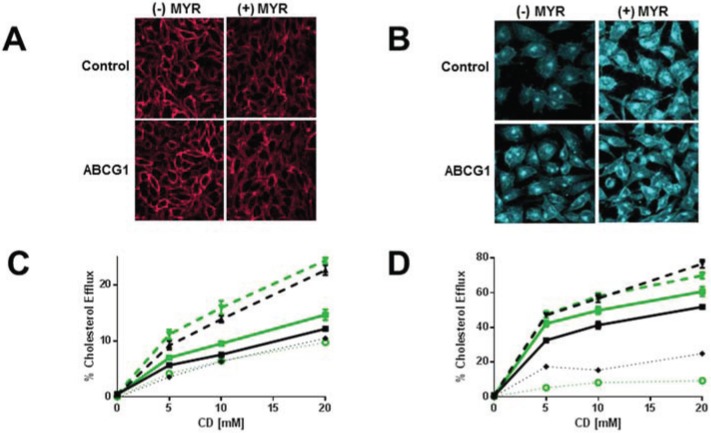

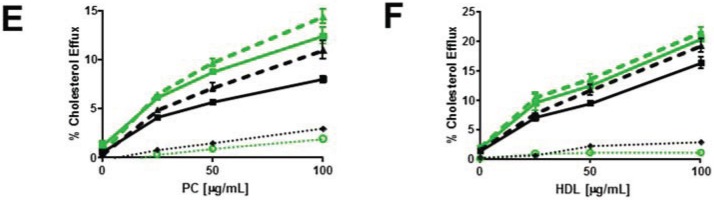
Myriocin reduces PM SM content and increases non-SM-associated cellular cholesterol pools and cholesterol efflux. (**A**) Myriocin reduces PM SM content. Control and ABCG1 cells pre-treated without myriocin ((−) MYR), or, with myriocin ((+) MYR) were immunostained with lysenin. (**B**) Myriocin increases cellular FC. Control and ABCG1 cells treated without ((−) MYR), or, with ((+) MYR) were stained with filipin. (**C**) Myriocin enhances CD-mediated PM FC efflux. Control (*black line*) and ABCG1 (*green line*) cells labeled with ^3^H-FC were pre-treated without or with myr (*solid and dashed lines*, respectively), and the percent of total cellular FC efflux CD (1 h at 4 °C) was determined. (**D**) Myriocin enhances CD-mediated cellular FC efflux. Control (*black*) and ABCG1 (*green*) cells labeled with ^3^H-FC were pre-treated without or with myr (*solid and dashed lines*, respectively), and the percent of total cellular FC efflux to CD (10 min at 37 °C) was determined. Myriocin enhances FC efflux to PC liposomes (**E**) and to HDL (**F**). (**E**, **F)**: *Black line*, *dashed black line*: untreated, and myr-treated control cells, respectively; *green line*, *dashed green line*: untreated, and myr-treated ABCG1 cells, respectively. *Dotted black* and *green lines* represent difference curves for control and ABCG1 cells with myriocin treatment, respectively. All values are expressed as mean ± S.D. Data shown is representative of at least three replicate experiments. Two-way ANOVA analyses using multiple comparisons revealed that in (**C**), all values are significantly different (*p* < 0.0001). In (**D**) all values are significantly different, except for control cells treated with myriocin *(black dashed line*) *vs*. ABCG1 cells treated with myriocin (green dashed line) at 5 and 10 mM CD (*p* >0.9999 and *p* = 0.9940, respectively). In (**E**), all values are significantly different (*p* < 0.0001) except for control cells (black solid line) *vs*. control cells treated with myriocin (black dashed line) at 25 µg/mL PC (*p* = 0.5727) and, ABCG1 cells (green solid line) *vs*. ABCG1 cells treated with myriocin (green dashed line) at 25 and 50 µg/mL PC liposomes (*p* = 0.9998 and *p* = 0.3189, respectively). In (**F**), all values for untreated control cells (solid black line) are significantly different from untreated ABCG1 cells (solid green line), *p* < 0.0001. The values for percent cholesterol efflux did not significantly differ at 25, 50 or, 100 µg/mL HDL for myriocin-treated control cells (dashed black line) *vs*. ABCG1 cells (solid green line) or myriocin-treated ABCG1 cells (dashed green line).

We next determined the effect of chronic reduction of cellular SM by inhibiting SM synthesis with myriocin, a serine palmitoyltransferase inhibitor [32], on ABCG1-mediated cholesterol efflux. As seen in Figure 6A, myriocin reduced cellular SM to a similar extent in both control and ABCG1 cells. Cytochemical analysis using filipin (Figure 6B) revealed that myriocin treatment increased filipin-accessible cholesterol at the PM and intracellular compartments of both control and ABCG1 cells, such that control and ABCG1 cells appear to have similar cholesterol content (Figure 6B). Myriocin increased ^3^H-cholesterol efflux to CD at 4 °C to a similar extent (~two fold) in both control and ABCG1 cells (Figure 6C). This finding suggests that myriocin-mediated depletion of PM SM increased the pool size of non-SM-associated PM cholesterol to a similar extent in ABCG1 and controls. The myriocin-induced increase far exceeded the ABCG1-mediated increase in PM cholesterol efflux in both control and ABCG1 cells. Myriocin also increased cellular ^3^H-cholesterol efflux to CD at 37 °C in both control and ABCG1 cells (Figure 6D). Interestingly, the myriocin-induced increase in cellular ^3^H-cholesterol efflux was greater in control cells (Figure 6D), and consequently, ^3^H-cholesterol efflux to CD at 37 °C was equivalent in control and ABCG1 cells.

We next investigated the effect of myriocin-mediated reduction of cellular SM levels on cellular cholesterol efflux to extracellular acceptors with a lipid surface. As seen in Figure 6E, reduction of cellular SM content increased ^3^H-cholesterol efflux to PC liposomes from both control and ABCG1 cells to a similar extent. HDL-mediated ^3^H-cholesterol efflux was similarly enhanced by treatment with myriocin. Thus, myriocin-mediated SM reduction appears to increase the amount of non-SM-associated-cholesterol in both control and ABCG1 cells, and thereby increase the total amount of cellular cholesterol available to efflux to CD, liposomes and HDL. Taken together, these findings indicate that both myriocin and ABCG1 increase CD-mediated cellular cholesterol efflux by the same mechanism, that is, expansion of the pool of non-SM-associated-cholesterol.

### 3.5. ABCG1 Confers Resistance to both Cholesterol-Dependent and Sphingomyelin-Dependent Toxin-Induced Cytolysis

**Figure 7:**
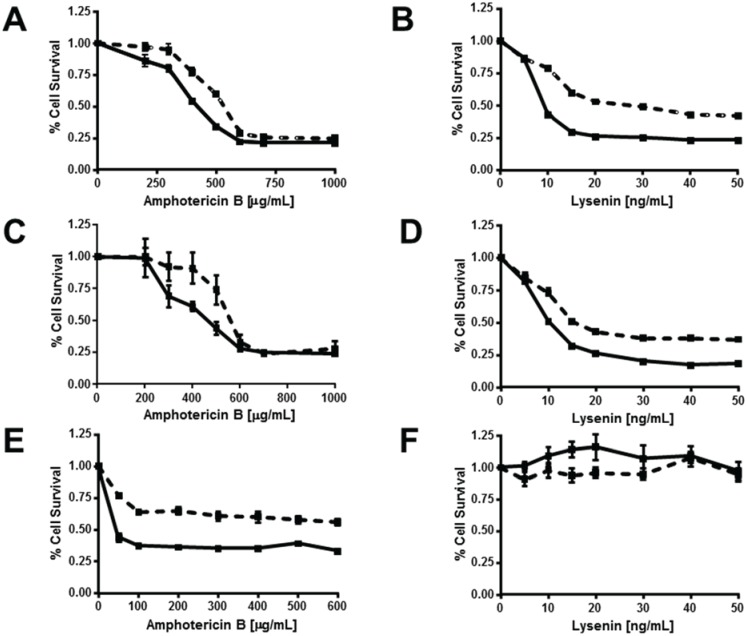
ABCG1 confers resistance to both amphotericin B and lysenin-mediated cytolysis. Control (*solid lines*) and ABCG1 cells (*dashed lines*) not pre-treated (**A**,**B**), or, pre-treated with exogenous SM (**C**,**D**), or, pre-treated with myriocin (**E**,**F**) were assayed for cell viability with varying doses of the FC-binding toxin amphotericin B (**A**,**C**,**E**), or the SM-binding toxin lysenin (**B**,**D**,**F**), as described in “Experimental Section.” Values are expressed as mean + S.E.M. Data shown is representative of at least three replicate experiments. Two-way ANOVA analyses using multiple comparisons revealed that ABCG1 expression significantly increased the percent Cell Survival at amphotericin B concentrations of (**A**) 200, 300, 400, and 500 µg/mL (*p* = 0.02, *p* < 0.0004, *p* < 0.0001, and *p* < 0.0001, respectively; (**C**) 200, 300, 400, and 500 µg/mL (*p* = 0.003, *p* < 0.0001, *p* < 0.0001, and *p* < 0.0001, respectively; (**E**) at all concentrations, *p* < 0.0001. Two-way ANOVA analyses using multiple comparisons revealed that ABCG1 expression significantly increased the percent Cell Survival at lysenin concentrations of 10–50 ng/mL (*p* < 0.0001) for (**B**) and (**D**). None of the values in (**F**) were significantly different (range *p* < 0.1954 to *p* < 0.9999).

We further explored the nature of ABCG1-induced alterations in the disposition of PM cholesterol and SM by monitoring cell death induced by the pore-forming cytotoxins amphotericin B and lysenin, which bind to cholesterol [33] and SM [34], respectively. These toxins induce cytolysis by a multi-step process that includes binding of toxin monomers to the cell surface, assembly of oligomers that, concomitant with membrane lipid organization, induce the formation of aqueous pores that increase membrane permeability and cause cell lysis [33,35]. Amphotericin B forms aqueous pores in ordered membrane microdomains enriched with cholesterol and SM [36,37], and such pores have been proposed to preferentially be localized at the phase boundary between L_o_ and L_d_ [33]. Lysenin can form pores in homogeneous SM-rich and cholesterol-rich L_o_-membrane regions indicating that the phase boundary is not necessary for lysenin pore formation [29,38]. Surprisingly, despite their increased plasma membrane cholesterol content (Figure 1, Figure 2 and Figure 3, Figure 5, Figure 6), ABCG1 cells were more resistant to amphotericin B-induced cytolysis compared to control cells (LD_50_ = 600 µg/mL *vs*. 400 µg/mL, respectively; Figure 7A). A similar paradoxical increase in amphotericin B resistance has been reported in a CHO cell cholesterol trafficking mutant with unaltered plasma membrane cholesterol content [20]. This increased resistance to amphotericin B cytolysis in ABCG1 cells is likely due to decreased pore formation by cholesterol-bound amphotericin B, as a result of ABCG1-induced alterations in membrane cholesterol organization. Interestingly, ABCG1 cells were also more resistant to lysenin-induced cytolysis compared to control cells (LD_50_ = 30 ng/mL *vs*. 10 ng/mL, respectively; Figure 7B). Since lysenin-binding to the PM assessed by immunostaining was similar in ABCG1 and control cells (Figure 1), as is the amount of SM-associated cholesterol (Figure 2), this finding suggests that ABCG1 alters the organization of SM in the plasma membrane such that lysenin pore-formation is inhibited. Enrichment of cells with exogenous SM had little effect on either amphotericin B- or lysenin-mediated cytolysis (Figure 7C,D *vs.* Figure 7A,B) in control or ABCG1 cells, suggesting that the expansion of SM-associated pools of cholesterol did not alter either SM or cholesterol organization, or, ABCG1-induced changes in SM or cholesterol organization.

This finding is consistent with the model where ABCG1, in the presence of exogenous SM, further expands SM-associated pools of cholesterol compared to controls, with concomitant ABCG1-enhanced conversion of L_o_-cholesterol to L_d_-cholesterolMyriocin-mediated reduction of cellular SM rendered both control and ABCG1 cells resistant to lysenin-mediated cytolysis (Figure 7F), suggesting that myriocin treatment decreased PM SM content below the threshold required for cytolysis in either cell type. In marked contrast, myriocin-mediated reduction of cellular SM (Figure 7E) increased the susceptibility of both control and ABCG1 cells to amphotericin B-mediated cytolysis. This finding is consistent with the increased non-SM-associated PM cholesterol observed in both cell types (Figure 6C). ABCG1 cells remained relatively resistant to amphotericin B-mediated cytolysis even with myriocin treatment, suggesting that ABCG1 still perturbed the organization of PM cholesterol, even with reduced levels of PM SM.

## 4. Conclusions

**Figure 8:**
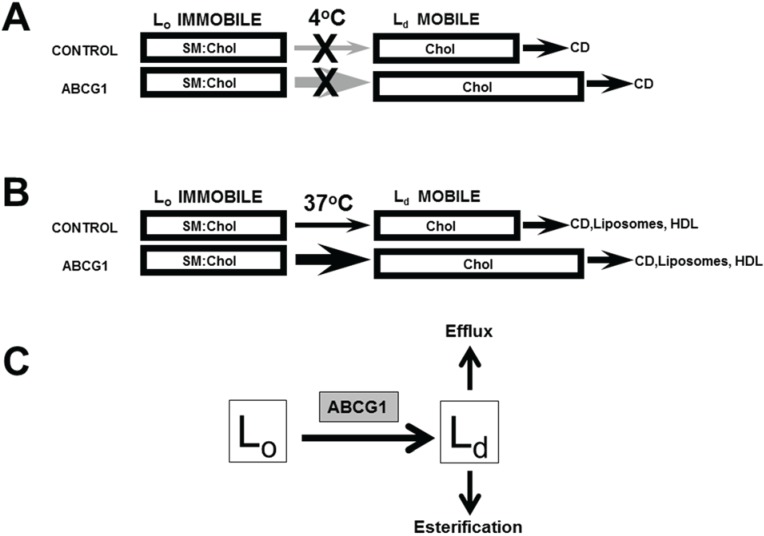
Model of ABCG1-mediated mobilization of cellular cholesterol for efflux and esterification. ABCG1 increases the flux of PM (**A**) and cellular (**B**) cholesterol (FC) between SM-associated FC in L_o_ membrane domains and non-SM-associated FC in membrane domains L_d_ pools. (**A**) At 4 °C, since FC movement from intracellular compartments to the PM as well as movement between L_o_ and L_d_ membrane domains at the PM is blocked, CD removes FC only from mobile L_d_ PM FC pools, which are expanded by ABCG1, as shown. (**B**) At 37 °C, CD, PC liposomes, and HDL remove mobile FC pools from the PM and intracellular compartments, mostly LE. Depletion of mobile FC pools results in the movement of FC from L_o_ to L_d_ membrane domains and subsequent removal by extracellular lipid acceptors from mobile pools. ABCG1 enhances movement of FC between L_o_ and L_d_ membrane domains in the PM and LE, thereby increasing FC efflux. (**C**) ABCG1 residing at the phase boundary between L_o_ and L_d_ membrane domains at the PM and LE appears to increase the rate of flux of cholesterol between L_o_ and L_d_ membrane domains, and therefore the size of the pool available to desorb to acceptors and in addition, may increase the chemical potential of L_d_ cholesterol. The mobile pools of FC in L_d_ membrane domains are available for removal on either side of the membrane lipid bilayer, by extracellular acceptors (efflux), or, by cytosolic carrier proteins, which promote movement to intracellular sites of esterification.

Using a heterologous expression system, we have previously shown that GFP-tagged human ABCG1 residing on the PM and LE enhances cellular cholesterol efflux to lipoproteins and liposomes, but not to apoA-I, thereby validating this model system for ABCG1 localization and function [1]. We further showed that ABCG1 at the PM mobilizes PM cholesterol and ABCG1 in LE/LYS generates mobile pools of cholesterol that can traffic by both vesicular and non-vesicular pathways to the PM [1]. We have used this heterologous expression system to gain further insights into the site(s) and mechanism of function of the human ABCG1 transporter. Our present study confirms that ABCG1-mobilized cholesterol rapidly cycles between the PM and LE/LYS, and revealed that ABCG1-mediated alterations in membrane cholesterol and SM organization mobilizes non-SM-associated cholesterol on the PM and LE for efflux and esterification (Figure 8).

ABCG1 expression increased PM and intracellular pools of cholesterol available to efflux to CD, PC liposomes and HDL. ABCG1 did not alter the amount or trafficking of GM_1_ nor the content of either SM or SM-associated-cholesterol in L_o_ PM membrane domains. Expansion of non-SM-associated pools of cellular cholesterol via SMase-mediated digestion of SM, or, myriosin-mediated inhibition of SM synthesis, mimicked ABCG1-mediated expansion of mobile, non-SM-associated (L_d_) pools of cellular cholesterol. Recent molecular dynamic simulations suggests that cholesterol effluxes to extracellular acceptors from L_d_ membrane domains and, that cholesterol migrates to L_d_ from L_o_ as cholesterol is depleted from L_d_ [11]. Our combined studies of SMase and myriocin-mediated reduction of SM-associated-cholesterol suggest the rate or amount of displacement of cholesterol from cholesterol from L_o_ to L_d_ may depend on the size of the SM-associated pool, and, that ABCG1 displaces only a fraction of the total pool of cholesterol that potentially may be mobilized from L_o_ to L_d_.

Interestingly, our studies suggest that a lipid phase boundary, not SM, may be required for ABCG1 function, because ceramide generated by SMase digestion of membrane SM was sufficient to sustain ABCG1-mediated cholesterol efflux. SMase-mediated hydrolysis of PM SM in cholesterol-enriched L_o_ domains generates ceramide in L_o_ domains. Ceramide, which is soluble in cholesterol-rich membranes, displaces cholesterol from cholesterol-rich L_o_ domains to cholesterol-poor L_d_ domains [39,40]. Cholesterol in L_d_ domains has increased chemical potential, so by generating ceramide, SMase facilitates cholesterol efflux by driving cholesterol movement from L_o_ domains to L_d_ domains. As ceramide equilibrates between cholesterol-rich and cholesterol-poor membranes it becomes insoluble and coalesces into gel-like domains (ceramide platforms) [41] that are resistant to cold Triton X-100 extraction [40].

Consistent with these previous observations, our current findings suggest that ABCG1-mediated changes in membrane lipid organization may facilitate the equilibration of SMase-generated ceramide between L_o_ and L_d_, thereby increasing the rate of ceramide-platform formation. This model is consistent with the observed formation of non-SM detergent-resistant membrane in SMase-treated ABCG1, but not SMase-treated control cells. In the absence of AGCG1, control cells presumably form less ceramide platforms and possess a larger pool of soluble ceramide. If soluble ceramide competes with, or otherwise inhibits cholesterol for transfer to a lipid surface, then SMase-treatment would decrease cholesterol efflux to PC liposomes and HDL in control cells, as we have observed. Consistent with this, in marked contrast to SMase, in control cells myriocin-induced reduction of PM SM (in the absence of ceramide generation) increased both the cellular pool of non-SM-associated cholesterol as well as cellular cholesterol efflux to PC liposomes and HDL. Together these findings suggest that SMase-generated ceramide may interfere with the transfer of non-SM-associated cholesterol to a lipid surface, and that ABCG1-mediated alterations in membrane lipid organization are able to overcome this ceramide-mediated inhibition.

Our studies of the effect of expansion of SM-associated pools of cholesterol with exogenous SM on ABCG1-mediated cholesterol efflux provided additional insights into ABCG1 functionality. In the absence of ABCG1 expression, exogenously supplied SM expanded PM pools of SM and SM-associated cholesterol, and unexpectedly, cellular pools of non-SM-associated-cholesterol as well. This finding suggests that the L_o_-cholesterol (SM-associated) and L_d_-cholesterol (non-SM-associated) are in dynamic equilibrium, such that expansion of the L_o_-cholesterol pool of cholesterol by increasing PM SM, also expands L_d_-cholesterol. In ABCG1 cells in the presence of exogenous SM, the pre-existing pool of excess non-SM-associated-cholesterol further expanded SM-associated pools of cholesterol compared to controls and, the increased ABCG1-mediated conversion of L_o_-cholesterol (SM-associated) to L_d_-cholesterol (non-SM-associated) even further expanded the pool of L_d_-cholesterol (non-SM-associated) cholesterol available for efflux (Figure 8).

We propose the following model for ABCG1 functionality outlined in Figure 8, wherein the transporter-induced redistribution of a single lipid species destabilizes the bilayer, and the subsequent re-organization to a lower membrane energy state generates the “effector” membrane domain. Briefly in this model (Figure 8), ABCG1 resides at the phase boundary between L_o_ and L_d_. ABCG1 binding to cholesterol and SM at the boundary stimulates ABCG1 ATP-ase activity. ABCG1 then redistributes a yet-to-be characterized lipid across the bilayer, creating an unstable energized state that is resolved by a redistribution of additional lipids across the bilayer, thereby altering the organization of cholesterol and SM in the membrane, and consequent displacement of cholesterol from L_o_ and L_d_. Precedent for this model is provided by the recent report that the transporters ABCA1 and ABCA7 export phophatidylcholine and phosphatidylserine, respectively, across membranes, whereas ABCA4 imported phophatidylethanolamine across membranes, when reconstituted into liposomes using fluorescent lipid analogues [42]. Additional support is provided by the proposed induction of transbilayer motion of other bilayer lipids together with lipid scrambling when ceramide is asymmetrically generated across the lipid bilayer, as with SMase digestion of SM [43]. Support for the localization of ABCG1 at the phase boundary between L_o_ and L_d_ membrane domains is provided by the our observation that ABCG1 is detergent-soluble, and that the ATPase activity of human ABCG1 in liposomes is stimulated by SM and cholesterol at concentrations lower than that required for L_o_ formation [4].

Since cholesterol rapidly cycles between L_o_ and L_d_ [8], ABCG1 may increase the efflux of cholesterol from L_d_ membrane regions simply by transiently increasing the available desorbable pool. Moreover, ABCG1-mediated alterations in membrane lipid organization in L_d_ membrane domains may additionally increase the chemical potential of cholesterol in L_d_ membrane domains compared to cholesterol in control cell L_d_ membrane domains. Both of these potential mechanisms are consistent with the findings by Sankaranarayanan *et al*. [31] that ABCG1 increases cell membrane free cholesterol pools available for efflux and its rate of desorption into the aqueous phase. Similarly, given the inherent increased rate of cholesterol flip-flop in L_d_ compared to L_o_ domains [44], the ABCG1-mediated increase of cholesterol available to desorb from cytosolic membrane leaflets may result from an increase in the size of the available pool of L_d_ cholesterol and/or increased chemical potential.

Taken together, our studies herein and in our previous report [1] suggest that the pool of ABCG1-activated L_d_ PM cholesterol can (i) be internalized as an endocytic membrane component and traffic along the endocytic pathway, (ii) be removed from the outer leaflet of the PM bilayer by an extracellular acceptor (HDL, LDL, PC liposome, CD), or, (iii) be removed from the inner leaflet of the PM or LE membrane by a cytoplasmic cholesterol acceptor protein and relocate to intracellular sites of esterification (Figure 8). In addition, we have previously shown that ABCG1 in LE mobilizes LE cholesterol for efflux via a non-vesicular pathway to the PM [1]. Thus, ABCG1 mobilizes potentially cytotoxic pools of non-SM-associated cholesterol into cytoprotective efflux and esterification pathways.

Finally, our model of ABCG1-induced alterations in membrane lipid organization is also consistent with the reported functionality of ABCG1 in specialized intracellular vesicles, namely, pancreatic β-cell secretory granules [45], and type II pneumocyte lamellar bodies [46], wherein membrane cholesterol is known to play specific roles in the formation and regulated secretion of both types of vesicles. Future studies will likely elucidate the downstream effector proteins and lipids that are modulated by ABCG1-mediated changes in membrane lipid organization.

## Supplementary Files

Supplementary File 1
