# Supplementary material for: The Human ABCG1 Transporter Mobilizes Plasma Membrane and Late Endosomal Non-Sphingomyelin-Associated-Cholesterol for Efflux and Esterification
Source: Biology (Basel). 2014 Dec 4;3(4):866–91. doi: 10.3390/biology3040866 (PMC4280515; doi:10.3390/biology3040866)

Supplement materials

Figure S1. ABCG1-GFP Thresholding. The GFP fluorescence was exceeding low compared to the robust “green” BODIPY-SM signal and, the contribution of GFP to the “green” fluorescence was essentially eliminated by adjusting the gain such that the GFP fluorescent signal from ABCG1 cells that were not labeled with BODIPY-SM was essentially eliminated, as shown below.

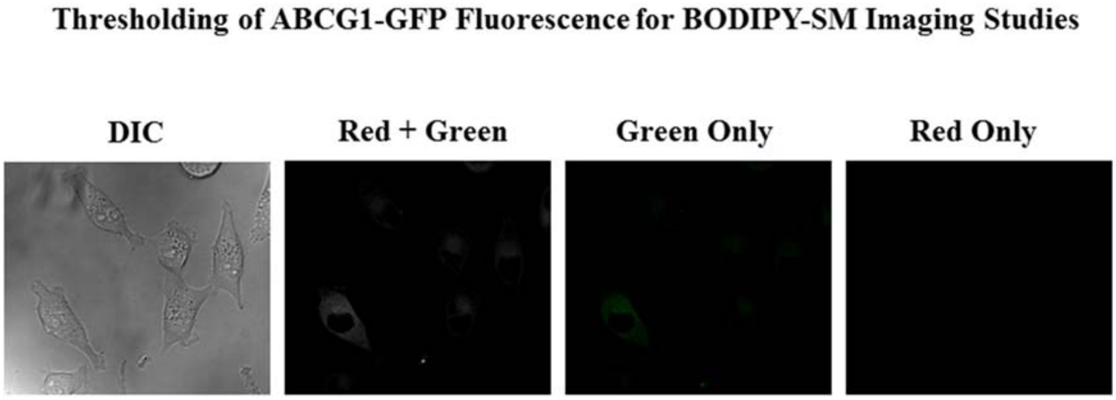

Figure S2. Incorporation of BODIPY-SM into the plasma membrane (PM) of control and ABCG1 cells. (**A**) Gallery showing SM fluorescence monitored in the “Red + Green”, “Green Only” and, “Red Only” emission channels. (**B**,**C**) Ratio fluorescence imaging of BODIPY-SM in control and ABCG1 cells. (**B**) Normalized emission spectra for ABCG1 and control cell PM fluorescence. Note that the intensity of the red fluorescence (>600 nm) for ABCG1 cells is increased ~2-fold compared ro control cells. (**C**) Note that in ABCG1 cells the the red/green fluorescence intensity is increased ~ 2-fold compared to control cells.

A

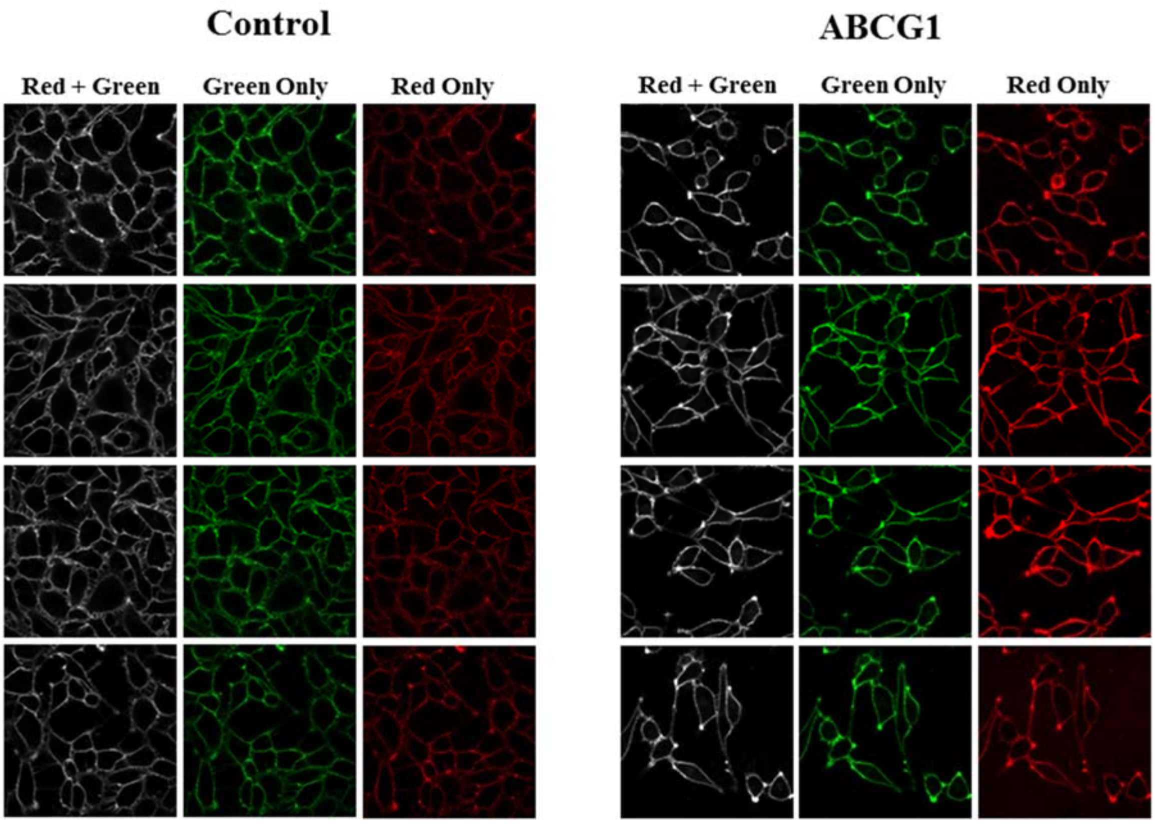

Figure S2. *Cont.*

B

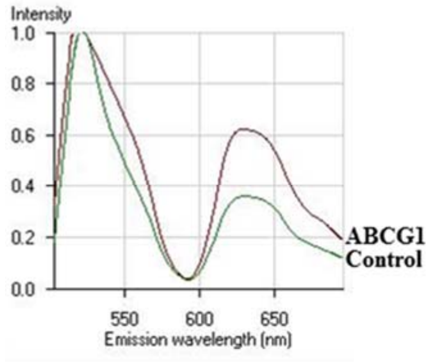

C

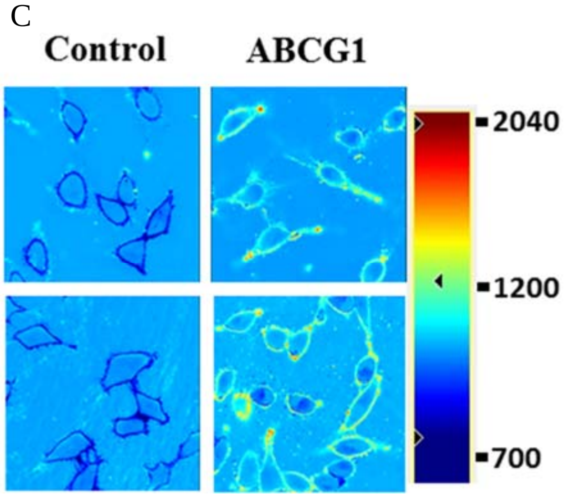

Figure S3. Trafficking of PM-derived BODIPY-SM in control and ABCG1 cells. (**A**) Gallery showing SM fluorescence monitored in the "Red + Green", "Green Only" and, "Red Only" emission channels. (**B**,**C**) Ratio fluorescence imaging of BODIPY-SM in control and ABCG1 cells.

A

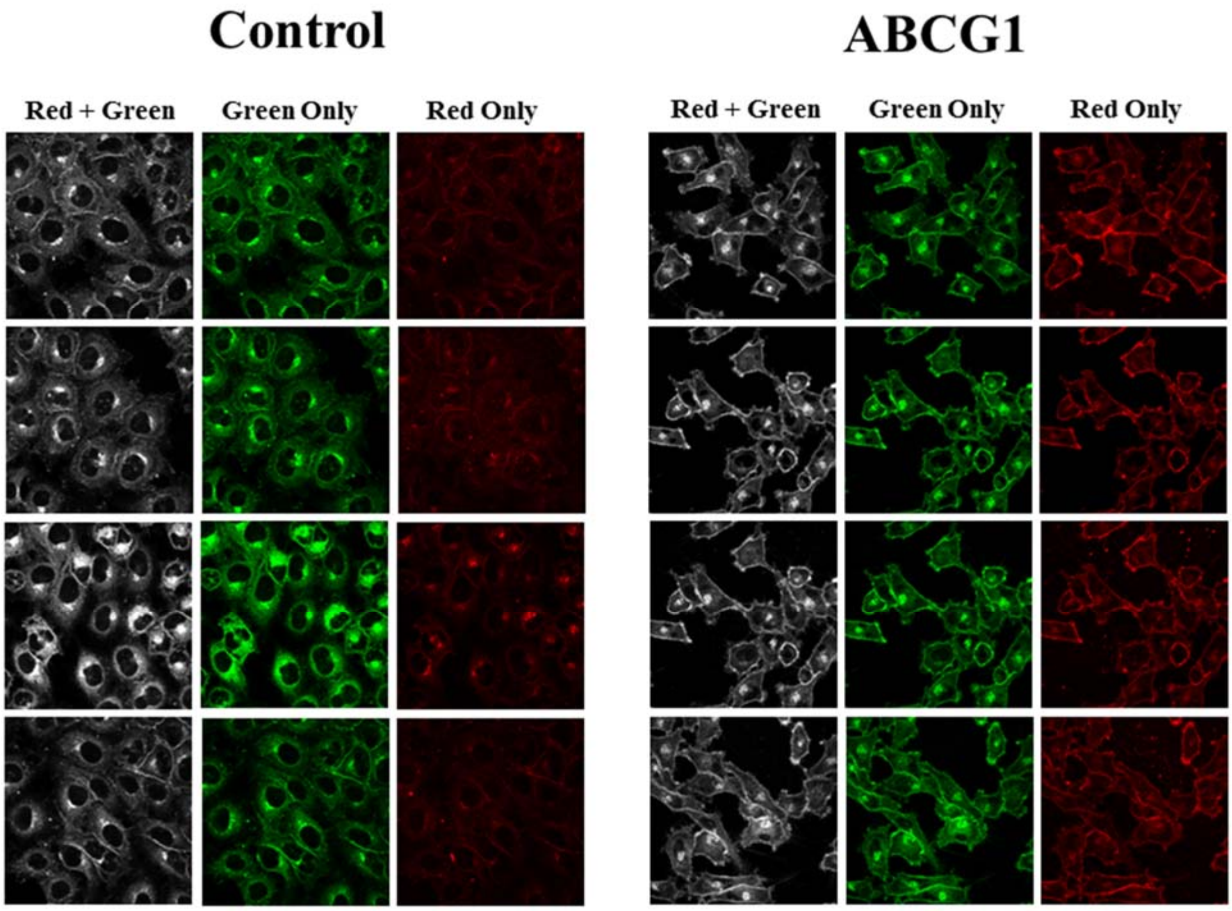

Figure S3. *Cont.*

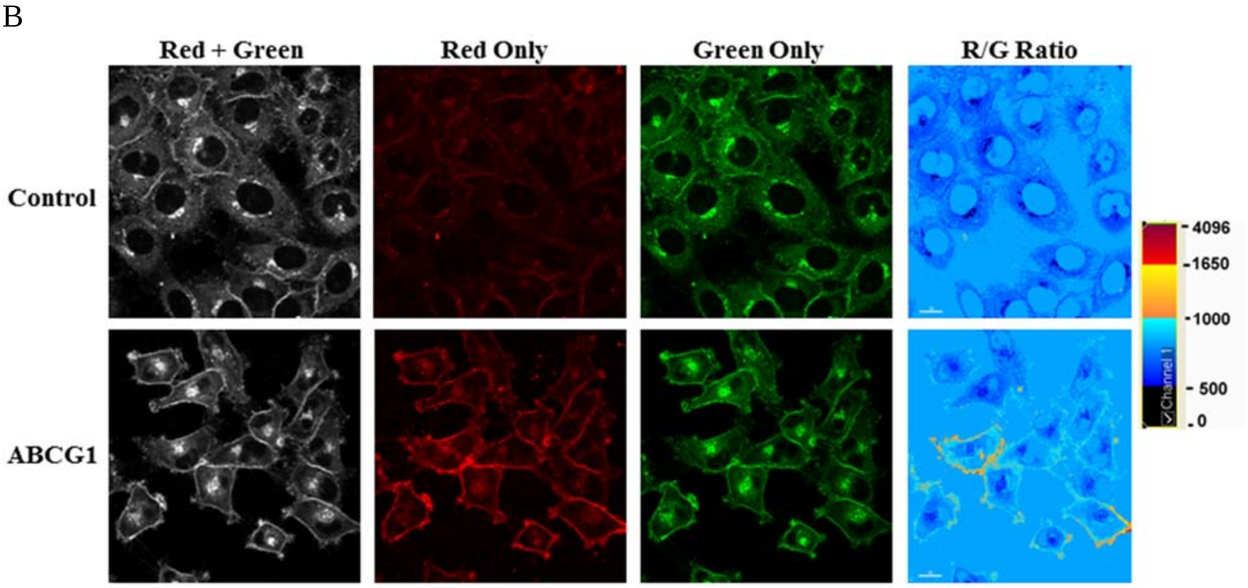

Figure S4. Rapid removal of BODIPY-SM from the PM and late endosomes/lysosomes of ABCG1 cells. Gallery showing SM fluorescence monitored in the "Red + Green", "Green Only" and "Red Only" emission channels in control and ABCG1 cells.

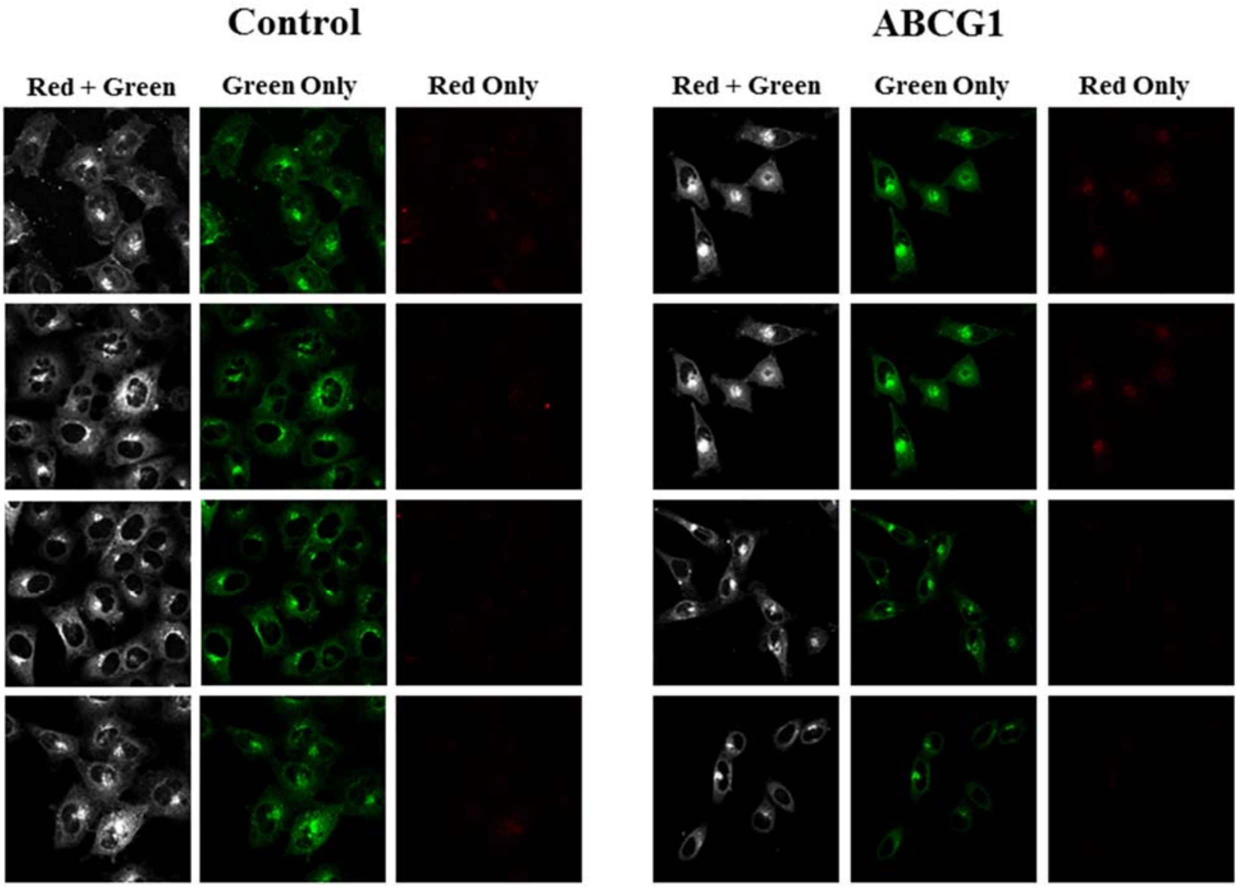

Figure S5. GM_1 trafficking in control and ABCG1 cells. GM_1 trafficking was monitored using cholera toxin B (CtxB), a GM_1-specific probe. Living control and ABCG1 cells were incubated with 100 ng/mL Alexa594-tagged CtxB in AMEM/0.1% BSA on ice for 20 min, and then chased in AMEM buffer medium supplemented with 10% fetal bovine serum, 2 mM glutamine, 100 IU/mL of penicillin, and 100 µg/mL streptomycin at 37 °C for 0, 10, 20, or, 60 min, fixed, and washed before imaging. Note that the intensity and distribution of CtxB is similar in control and ABCG1 cells at all times.

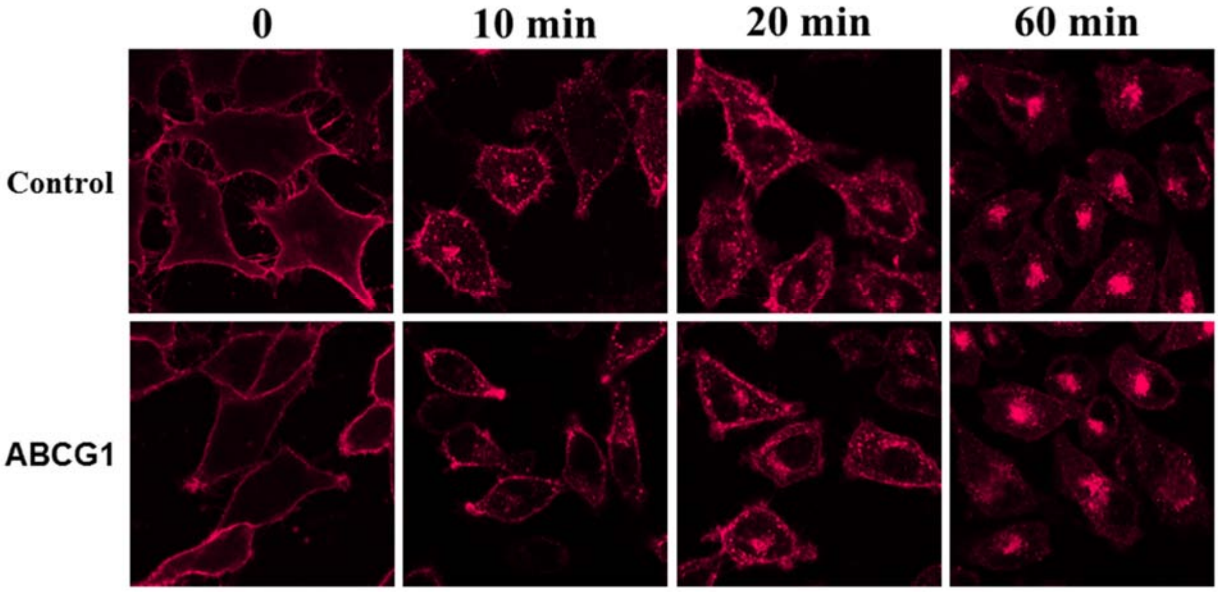

Figure S6. ABCG1-GFP alters cholesterol detergent solubility. Gallery of images.

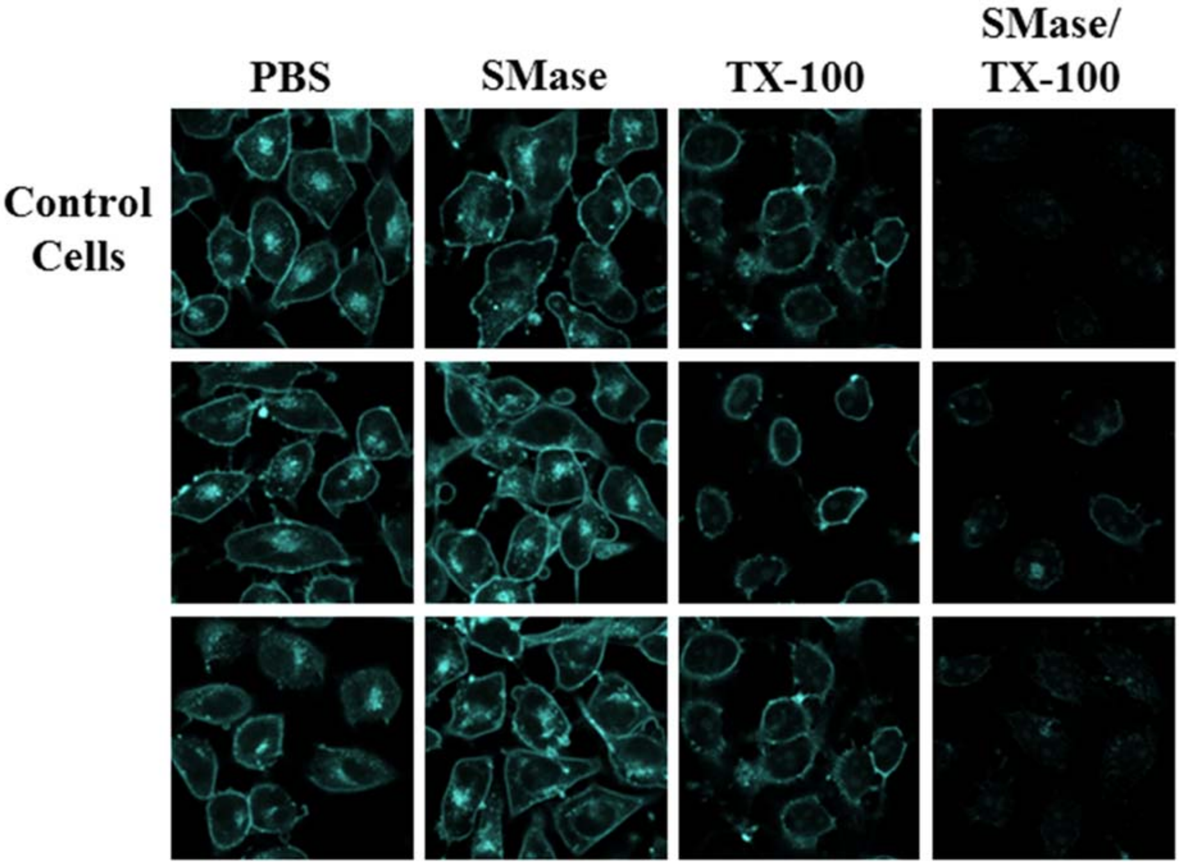

Figure S6. *Cont.*

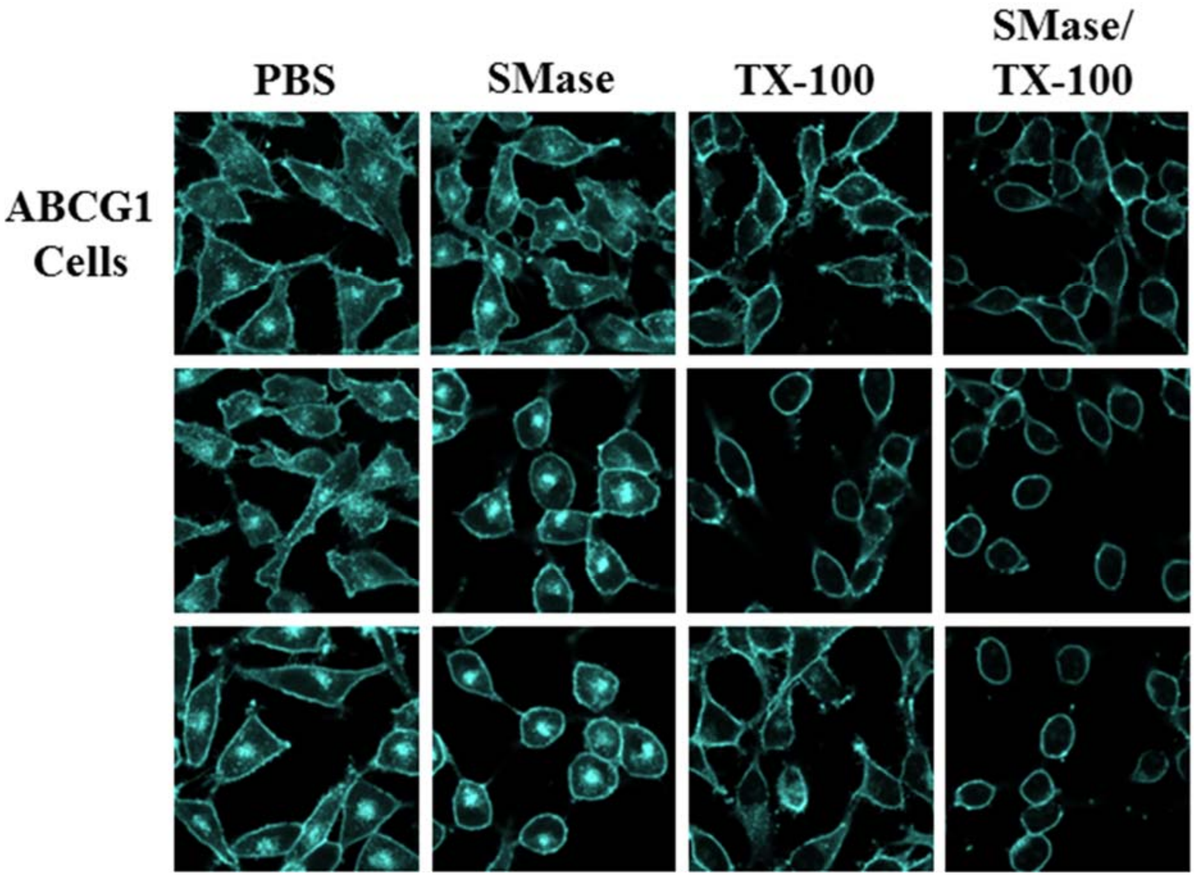

Figure S7. Cold CD depletes cholesterol from the PM from living cells. Gallery of images.

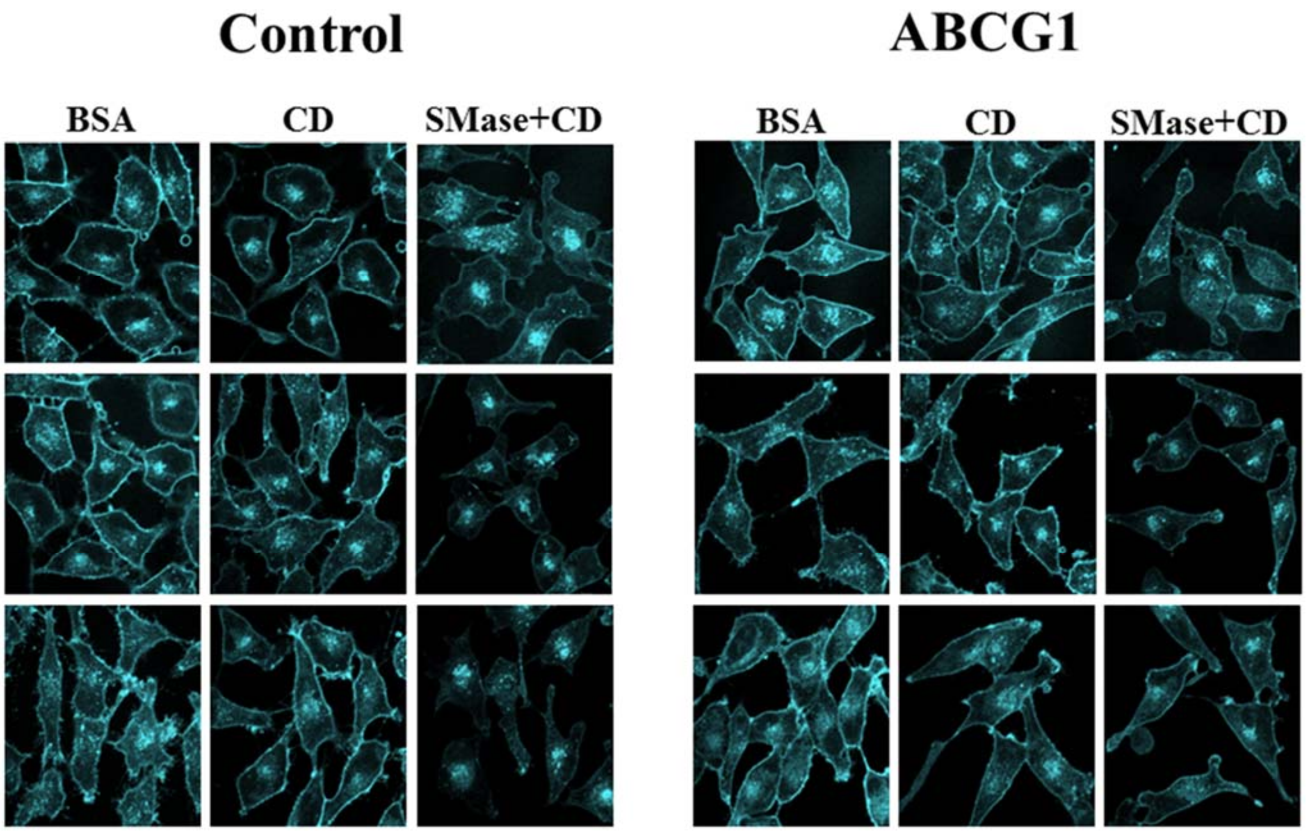

Figure S8. Warm CD depletes cholesterol from the PM and intracellular compartments of living cells. Gallery of images.

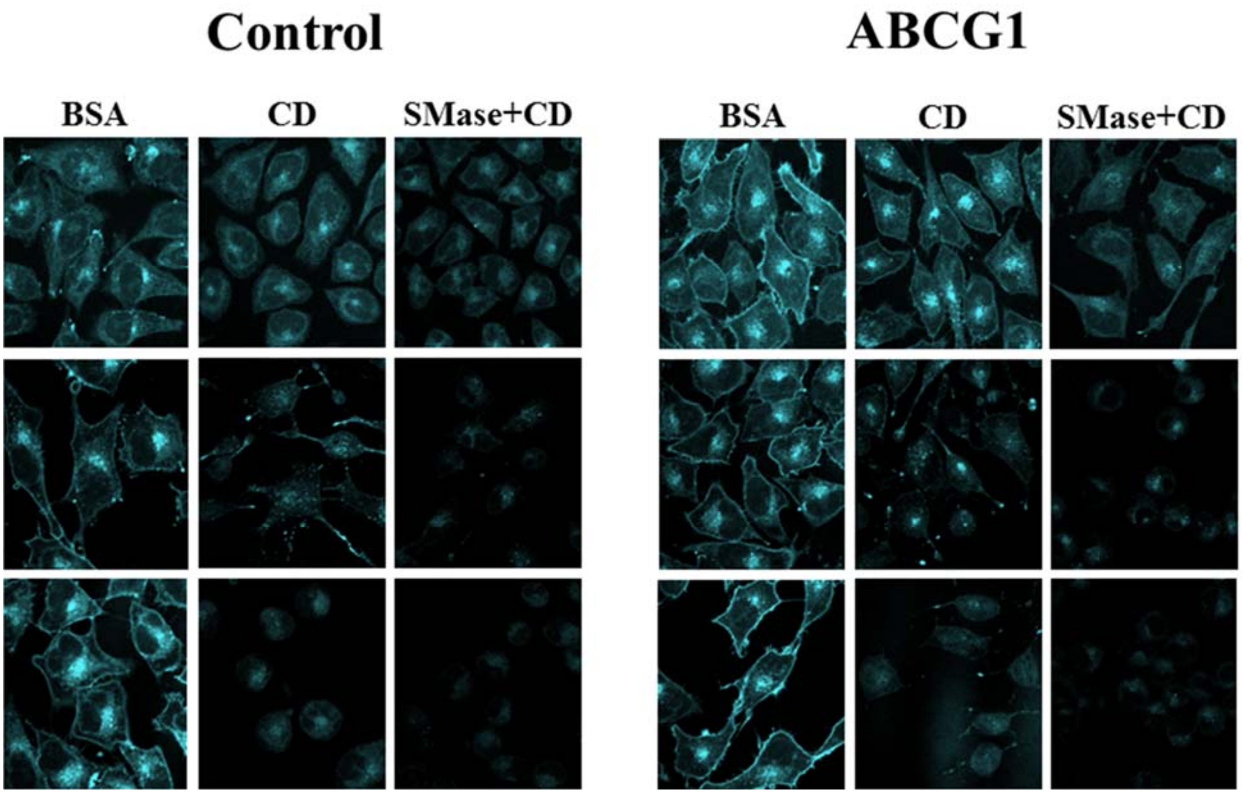

Figure S9. Gallery of confocal microscopic images of Nile Red stained control and ABCG1 cells.

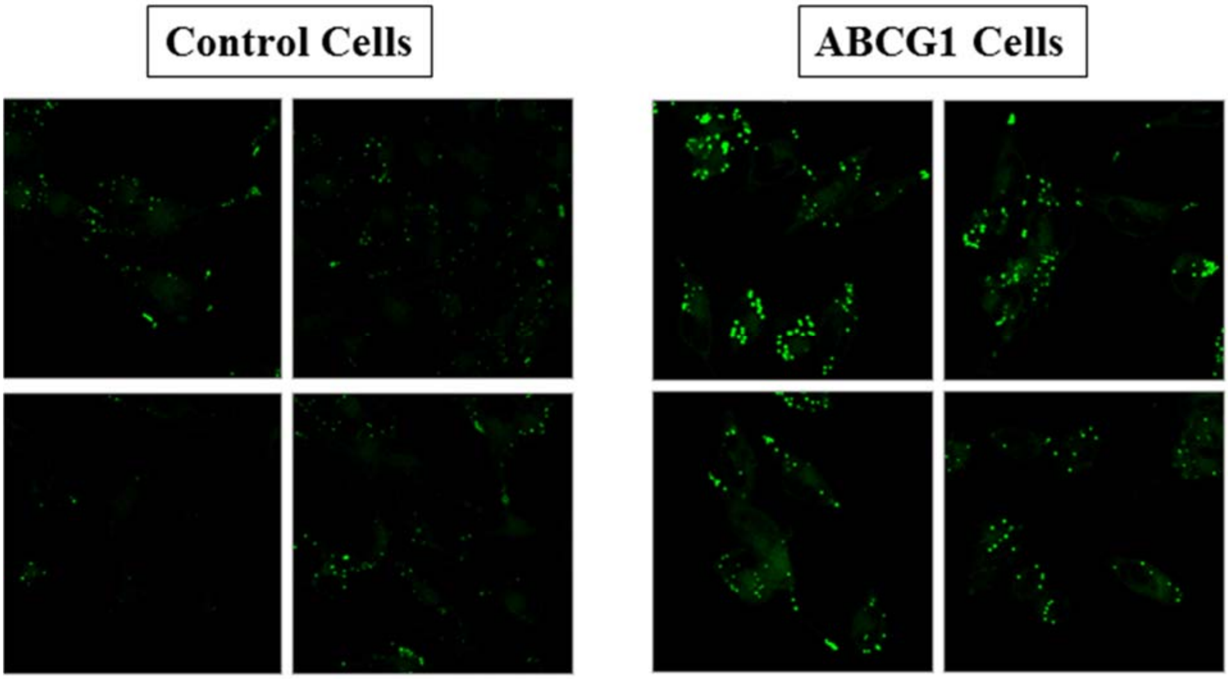

Supplement: Supplementary File 1 [file biology-03-00866-s001.pdf]
